# Biphenyl Ether Analogs Containing Pomalidomide as Small-Molecule Inhibitors of the Programmed Cell Death-1/Programmed Cell Death-Ligand 1 Interaction

**DOI:** 10.3390/molecules27113454

**Published:** 2022-05-27

**Authors:** Shabnam Shaabani, Louis Gadina, Ewa Surmiak, Zefeng Wang, Bidong Zhang, Roberto Butera, Tryfon Zarganes-Tzitzikas, Ismael Rodriguez, Justyna Kocik-Krol, Katarzyna Magiera-Mularz, Lukasz Skalniak, Alexander Dömling, Tad A. Holak

**Affiliations:** 1 Department of Drug Design, University of Groningen, 9713 AV Groningen, The Netherlands; s.shaabani@rug.nl (S.S.); zefeng.wang@rug.nl (Z.W.); bidong.zhang@rug.nl (B.Z.); roberto.butera@gmx.de (R.B.); tryfon.zarganis-tzitzikas@cmd.ox.ac.uk (T.Z.-T.); 2 Faculty of Chemistry, Jagiellonian University, 30-387 Krakow, Poland; louis.gadina@gmail.com (L.G.); ewa.surmiak@uj.edu.pl (E.S.); ismael.rodriguez@doctoral.uj.edu.pl (I.R.); justyna.kocik@doctoral.uj.edu.pl (J.K.-K.); k.magiera@uj.edu.pl (K.M.-M.); lukasz.skalniak@uj.edu.pl (L.S.)

**Keywords:** PD-1/PD-L1, small-molecule inhibitors, immune checkpoint blockade

## Abstract

New biphenyl-based chimeric compounds containing pomalidomide were developed and evaluated for their activity to inhibit and degrade the programmed cell death-1/programmed cell death- ligand 1 (PD-1/PD-L1) complex. Most of the compounds displayed excellent inhibitory activity against PD-1/PD-L1, as assessed by the homogenous time-resolved fluorescence (HTRF) binding assay. Among them, compound **3** is one of the best with an IC_50_ value of 60 nM. Using an ex vivo PD-1/PD-L1 blockade cell line bioassay that expresses human PD-1 and PD-L1, we show that compounds **4** and **5** significantly restore the repressed immunity in this co-culture model. Western blot data, however, demonstrated that these anti-PD-L1/pomalidomide chimeras could not reduce the protein levels of PD-L1.

## 1. Introduction

The discovery of immune checkpoint proteins (ICP) initiated therapies that activate the organism′s immune system to fight cancer, a strategy which is now known as immune oncology [1,2,3,4,5,6,7,8]. The most important ICPs are cytotoxic T lymphocyte-associated antigen 4, programmed cell death protein 1 (PD-1), and programmed cell death ligand 1 (PD-L1) [3,4,8]. In normal healthy tissue, the PD-1/PD-L1 pathway serves as a negative regulator of T cells and as such helps to maintain control of inflammation, prevents self-aggression, and serves as an adaptive arm of the immune system in pregnancy, tissue allographs, etc. [9,10,11,12]. Tumors hijack the checkpoint control system by producing PD-L1 and turn down the T cell response. Blocking PD-L1 or PD-1 allows for the T cell killing of tumor cells. These findings were recognized in the 2018 Nobel Prize in Physiology and Medicine to James P. Allison and Tasuku Honjo for the discovery of CTLA-4 and PD-1 as negative immune regulation targets for cancer therapy.

Cancer immunotherapy is currently based on monoclonal antibodies such as, for example, nivolumab, pembrolizumab, or atezolizumab [8]. There is no doubt that the treatments based on these biologicals have produced excellent results [13], however, mAbs show considerable side effects, which are persistent due to the long elimination half-time, their susceptibility for degradation, and low permeability [14,15]. Promising results have recently been reported for low-molecular-weight inhibitors such as peptides, small molecules, peptidomimetics, macrocycles which aim at the PD-1/PD-L1 interaction [16,17,18]. Worth mentioning is the fact that although there is a large variety of these compounds, only a few of them progressed into clinical trials with no clear results reported until today.

Within the field of the small-molecule inhibitors for the PD-1/PD-L1 system, the first and the leading breakthrough were the compounds based on the biphenyl scaffold reported by Bristol Myers Squibb [16,19]. Since then, biphenyl core structures are the predominant class, highly developed compounds, with affinity to PD-L1 reaching up to the nanomolar range; however, they still lack matching activity of the antibodies in in vitro cancer cell assays [18,20]. In general, designing small-molecule inhibitors of the PD-L1/PD-1 protein–protein interaction (PPI) is challenging because the PD-1/PD-L1 interaction interface creates a long, hydrophobic, and relatively shallow surface on PD-L1 [21,22,23].

In this study, we would like to present the synthesis and characterization of a series of PD-L1 dual inhibitors which utilize the E3 ubiquitin ligase cereblon inhibitor (pomalidomide) combined with either known biphenyl inhibitors (BMS-8 and BMS-1166) and the structures based on our own scaffolds [24]. This strategy allows for the blockade of the PD-1/PD-L1 immune checkpoint, but also should provide anticancer effects related to the binding to cereblon.

## 2. Results and Discussion

### 2.1. Design and Synthesis

**Scheme 1:**
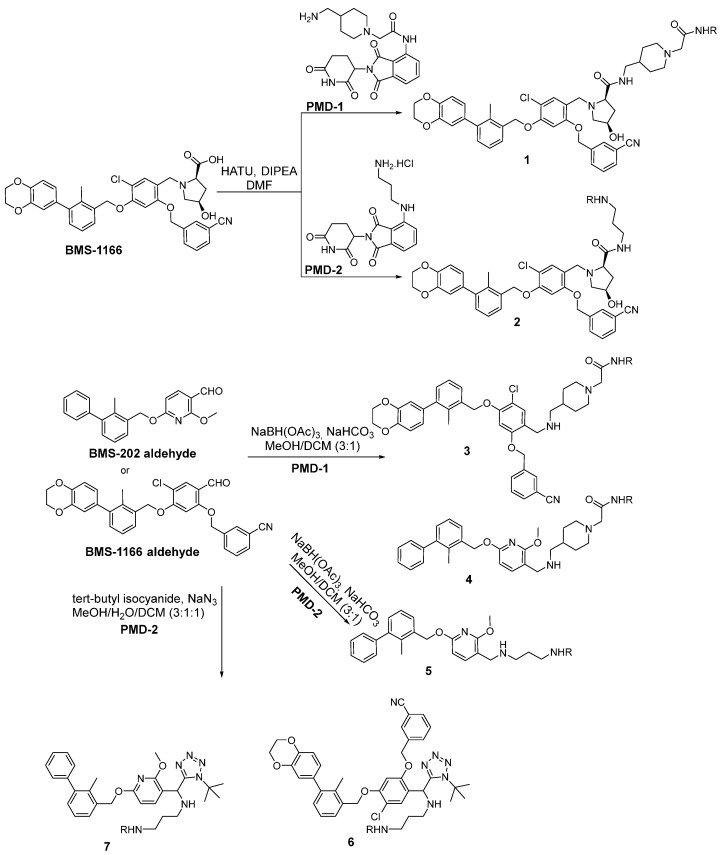
The synthetic pathways for the chimeras based on BMS-1166 and BMS-202. The R in all the products stands for the pomalidomide moiety.

**Scheme 2:**
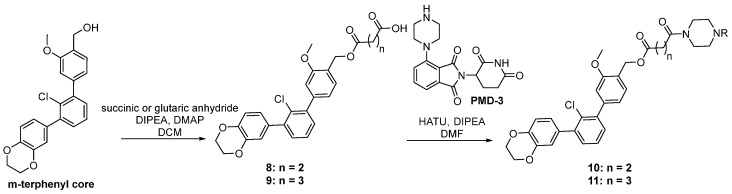
The synthetic pathways for the chimeras based on terphenyl PD-L1 inhibitors. The R in all the products stands for the pomalidomide moiety.

**Scheme 3:**
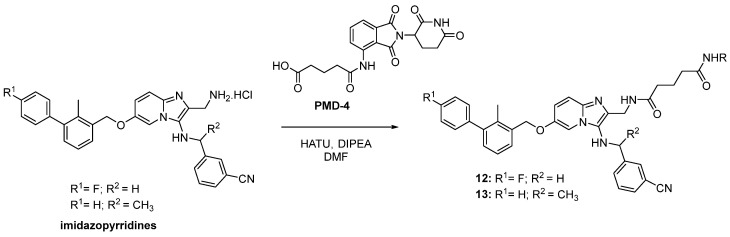
The synthetic pathways for the chimeras based on imidazopyridine PD-L1 inhibitors. The R in all the products stands for the pomalidomide moiety.

Inspired by recent findings concerning the effect of pomalidomide on PD-L1 induction [25] and postulated activity of PD-L1-targeting PROTACs [26,27], we designed several PD-L1–linker–pomalidomide synthetic constructs based on our recently published PROTAC and PD-L1 work [24,28]. The syntheses of the proposed inhibitors are shown in Scheme 1, Scheme 2 and Scheme 3 and comprise the known syntheses of the PD-L1 inhibitors BMS-1166 and BMS-202 [17,19,29,30], terphenyl [31,32] and imidazopyridines [24], which then were linked together with pomalidomide derivatives to form the desired chimeras. All the pomalidomide intermediates used in the synthesis were obtained according to the known protocols.

Biphenyl BMS-based chimeras were obtained either from a full-length carboxylic acid BMS-1166 by the HATU coupling with amine-derived pomalidomide (for compounds **1** and **2**) or from the corresponding BMS aldehydes via reductive amination (compounds **3**–**5**) and the Ugi tetrazole reaction (compounds **6** and **7**) with the same amine-derived pomalidomide (Scheme 1).

The syntheses of chimeras **10** and **11** were based on terphenyl PD-L1 inhibitors. An m-terphenyl scaffold was prepared using a known four-step protocol [31] and then combined with linkers through ester bond formation by the reaction with a suitable anhydride to form intermediates (**8** and **9**). These components bearing carboxyl acid functionality then reacted with amine-derived pomalidomide to form the final terphenyl chimeras **10** and **11** via the HATU coupling strategy (Scheme 2).

The biphenyl chimeras **11** and **12** containing imidazopyridines were prepared by means of the HATU coupling between the carboxylic acid-derived pomalidomide with amine PD-L1 inhibitors. The amine-barring PD-L1 inhibitors were obtained through the Groebke–Blackburn–Bienayme reaction as previously reported (Scheme 3) [24].

### 2.2. Binding Analysis

To validate the activity toward PD-L1 of our compounds, we carried out an HTRF (homogeneous time-resolved fluorescence) experiment at two concentrations of the ligand (5 µM and 0.5 µM) (Table 1). All the compounds inhibit the PPI at a higher concentration. The most potent are the compounds incorporating BMS-202 and BMS-1166 and a short aliphatic linker (compounds **3**–**5**). Surprisingly, the use of the full structure of BMS-1166 (with the solubility tag hydroxyproline) resulted in a drop of the activity (**1** and **2**). The least active BMS-based inhibitors were obtained after introducing the tetrazole ring into the linker (**6** and **7**). Furthermore, the chimeras based on our own scaffolds, imidazopyridines [24] and terphenyl [31], are significantly less active than the “best” compounds though similar to the elongated BMS-1166, **1**, and **2**. We determined the IC_50_ values for the best PD-L1 binding compounds, and these are shown in Table 1. The HTRF assay was validated on BMS-1166 yielding IC_50_ of 3.89 ± 0.19 nM (the 100% dissociated PD-1/PD-L1 complex at 5 and 0.5 µM concentrations). The pomalidomide fragment was tested in the same conditions as well, resulting in the 20.1% dissociated complex at 5 µM concentration and 11.4% at 0.5 µM. The small values may result from unspecific binding or protein precipitation during the assay. The lack of interaction between pomalidomide and PD-L1 was further proven by NMR titration (Figure S1).

**Figure 1:**
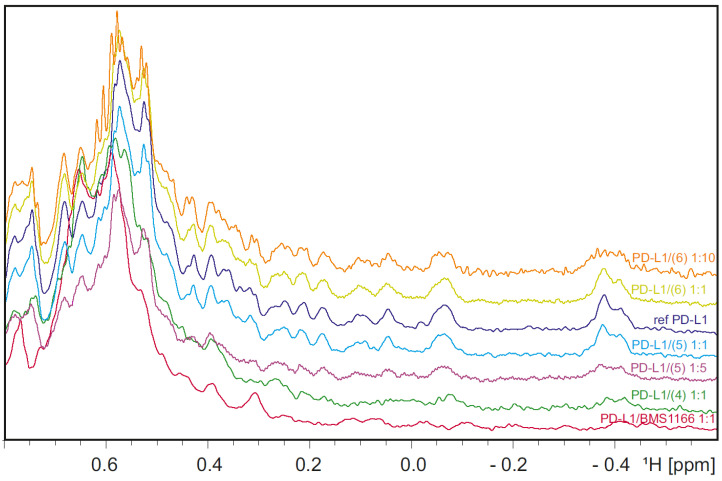
The aliphatic ^1^H NMR spectrum of the reference PD-L1 (blue) superimposed on the spectrum of the PD-L1 titrated with compounds **4** (green—protein–ligand molar ratio, 1:1), **5** (light blue—protein–ligand molar ratio, 1:1; purple—protein–ligand molar ratio, 1:5), **6** (yellow—protein–ligand molar ratio, 1:1; orange—protein–ligand molar ratio, 1:10), and the spectrum of PD-L1 titrated with a known PD-L1 inhibitor—BMS-1166 (red, protein–ligand molar ratio, 1:1).

Apart from the HTRF assay, we performed the NMR [33,34] binding analysis by recording the ^1^H spectrum of the PD-L1 protein titrated with compound **4** (Figure 1). The obtained results indicate that the tested compounds **4** and **5** bind to the PD-L1 protein. Furthermore, the broadening and despairing of the peaks suggest that the protein undergoes oligomerization induced by the binding to the ligand, which is visible for compound **4** at the protein–ligand ratio of 1:1 and 1:5 for compound **5**. Such behavior is characteristic of all the biphenyl-based inhibitors of PD-L1, like BMS-1166 [18,30]. We conclude that elongation of the PD-L1-binding scaffold, such as presented in compounds **4** and **5**, does not interrupt the binding to the PD-L1 protein. The inactive compound **6** does not present changes in the PD-L1 NMR protein spectra, although in the concentration of the protein–ligand ratio of 1:10, the intensity of the peaks is decreased due to massive protein precipitation visible in the test tube. Protein precipitation during titration was not observed for compounds **4**, **5**, and BMS-1166.

### 2.3. Cellular Activity Analysis

In order to explore the bioactivity of the synthesized compounds, a well-recognized immune checkpoint blockade (ICB) assay was performed [18,20,35,36]. In the assay, artificial T cells, the Jurkat effector cells (Jurkat ECs), i.e., the Jurkat T cells overexpressing PD-1 and luciferase-coding gene under the control of the TCR-inducible NFAT response element, are contacted with artificial antigen-presenting CHO/TCRAct/PD-L1 cells, as illustrated in [31]. The latter is the CHO cells equipped with the TCR activator molecule, which delivers activation of Jurkat ECs, and also the PD-L1 protein, which provides a negative signal towards the activation of Jurkat ECs. Upon the blockade of the PD-1/PD-L1 interaction, activation of Jurkat ECs is increased, as we have shown before for several classes of therapeutic antibodies and small molecules [18,20,35,36].

**Figure 2:**
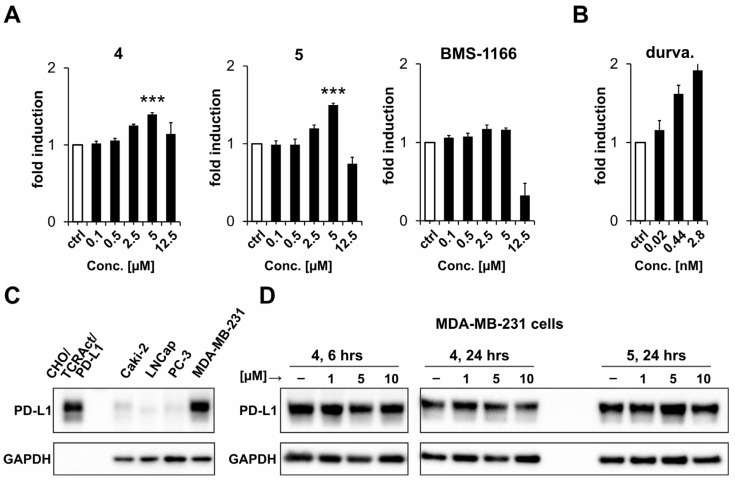
Bioactivity of the indicated compounds. (**A**,**B**) The activity was monitored with a cell-based immune checkpoint blockade (ICB) assay. Jurkat ECs were contacted with pre-seeded CHO/TCRAct/PD-L1 cells in the presence of the indicated concentrations of the compounds (**A**) or durvalumab (**B**) as a positive control. The cells were co-cultured for 6 h and the luminescence signal was measured following the addition of the Bio-Glo substrate. The graphs show fold induction calculated versus either DMSO-treated (for small molecules) or untreated (for durvalumab) control (ctrl) cells. The results are mean ± SD from three independent experiments. For statistical analysis, Student’s t-test was performed: ***, *p* < 0.001 vs. BMS-1166 treated cells. (**C**) Analysis of the expression of PD-L1 in four human cancer cell lines compared to PD-L1-overexpressing CHO/TCRAct/PD-L1 cells. (**D**) Analysis of the expression of PD-L1 in MDA-MB-231 cells treated with either **4** or **5** compounds for 6 or 24 h.

In this study, two of the best-performing molecules were used, i.e., **4** and **5**. Both compounds were able to increase the activation of Jurkat ECs, as also observed for a positive control antibody, durvalumab (Figure 2A,B). Importantly, at the concentration of 5 µM, both compounds presented significantly higher levels of the activation of Jurkat ECs than the BMS-1166 compound. At the same time, pomalidomide alone did not increase the activation of Jurkat ECs in the ICB assay (Figure S2). The PD-1/PD-L1 blockade evoked by **4** and **5** was not related to the downregulation of PD-L1 expression, as shown in Figure 2C,D for PD-L1-expressing MDA-MB-231 cells.

## 3. Conclusions

The results presented here demonstrate that the incorporation of the linker and pomalidomide into the structure of the BMS-202 molecule not only does not disturb its biological properties, but also increases its bioactivity. The presented chimeric compounds were able to bind to the human PD-L1 protein and dissociate the PD-1/PD-L1 complex in the HTRF assay. The PD-1/PD-L1 blockade induced by **4** and **5** was unrelated to the downregulation of PD-L1 expression in the PD-L1-expressing MDA-MB-231 cells. This is in clear contrast to previous reports which claimed PROTAC-like activity of similar pomalidomide/thalidomide-containing chimeras [26,27]. Of note, downregulation of PD-L1 expression was very limited in these studies and was only observed at considerably high concentrations (2.5–10 µM), which is unusual for successful PROTAC probes, which are known to be active in the pM–nM concentration range. In conclusion, the introduction of a CRBN ligand into BMS-202 enhances the blockade of PD-L1 in a cellular context but does not interfere with PD-L1 expression, possibly due to the disjoint localization of the two molecular targets (the extracellular domain of PD-L1 and the intracellular CRBN protein).

## 4. Materials and Methods

### 4.1. Synthesis

All the syntheses were performed using general procedures summarized in Scheme 1, Scheme 2 and Scheme 3 and as described below in detail. Reagents were obtained from commercial suppliers (Sigma Aldrich, St. Louis, MO, USA; ABCR, Karlsruhe, Germany; Acros Organics, Thermo Fisher Scientific, Geel, Belgium; Apollo Scientific, Stockport, Cheshire, UK; AK Scientific, Union City, CA, USA) and used without further purification unless otherwise noted. Anhydrous solvents were purchased from Sigma-Aldrich or Alfa-Aesar, anhydrous Et_2_O and THF were distilled from sodium benzophenone and stored over molecular sieves (4Å, 3–5 mm beads). Nuclear magnetic resonance spectra were recorded using Bruker Avance 500 or 600 spectrometers (^1^H NMR (500 MHz; 600 MHz), ^13^C NMR (126 MHz; 151 MHz)). Chemical shifts for ^1^H NMR were reported as d values and the coupling constants were in hertz (Hz). The following abbreviations were used for spin multiplicity: s = singlet, _br_s = broad singlet, d = doublet, t = triplet, q = quartet, quin = quintet, dd = double of doublets, ddd = double doublet of doublets, m = multiplet. Chemical shifts for ^13^C NMR were reported in d relative to the solvent peak. Thin-layer chromatography was performed on Sigma-Aldrich/Fluka precoated silica gel plates (0.20 mm thick, particle size 25 mm). Flash chromatography was performed on a Reveleris^®^ X2 Flash Chromatography using Grace^®^ Reveleris Silica flash cartridges. Chromatographic separations were carried out using an Acquity UPLC BEH (bridged ethyl hybrid) C_18_ column, 2.1 mm × 100 mm, and 1.7 µm particle size, equipped with an Acquity UPLC BEH C18 VanGuard precolumn, 2.1 mm × 5 mm, and 1.7 µm particle size. The column was maintained at 40 °C, and eluted under gradient conditions using from 95% to 0% of eluent A over 10 min at a flow rate of 0.3 mL min^−1^. Eluent A: water/formic acid (0.1%, *v*/*v*); eluent B: acetonitrile/formic acid (0.1%, *v*/*v*). The purity of all the final compounds determined using chromatographic LC–MS was > 95%. HRMS was carried out by the Laboratory for Forensic Chemistry, Faculty of Chemistry, Jagiellonian University, with a microOTOF-QII spectrometer using the ESI technique.

(2*R*,4*R*)-1-(5-chloro-2-((3-cyanobenzyl)oxy)-4-((3-(2,3-dihydrobenzo[b][1,4]dioxin-6-yl)-2-methylbenzyl)oxy)benzyl)-N-((1-(2-((2-(2,6-dioxopiperidin-3-yl)-1,3-dioxoisoindolin-4-yl)amino)-2-oxoethyl)piperidin-4-yl)methyl)-4-hydroxypyrrolidine-2-carboxamide (**1**)

HATU (1.2 equiv.) and N,N′-diisopropylethylamine (seven equiv.) were added to a solution of BMS-1166 (0.1 mmol) in 1 mL DMF and stirred. After 10 min, amine-derived pomalidomide hydrochloride salt (PMD-1, 0.1 M in DMSO) was added to the reaction. After 12 h, the reaction mixture was lyophilized (to remove the solvent) and the crude substance was purified by flash column chromatography giving compound **1** as white powder with 12% yield. The amine-derived pomalidomide and BMS-1166 used in the synthesis were obtained according to known protocols [28,30].

^1^H NMR (500 MHz, CDCl_3_) (mixture of diastereomers): δ, 11.37–11.28 (m, 1H), 8.89–8.79 (m, 1H), 7.75–7.71 (m, 1H), 7.69 (t, *J* = 7.9 Hz, 1H), 7.66–7.57 (m, 2H), 7.55–7.44 (m, 2H), 7.41–7.33 (m, 2H), 7.30 (d, *J* = 5.7 Hz, 1H), 7.24–7.20 (m, 2H), 6.91 (d, *J* = 8.2 Hz, 1H), 6.81 (d, *J* = 2.0 Hz, 1H), 6.79–6.73 (m, 1H), 6.55 (d, *J* = 11.1 Hz, 1H), 5.12–5.05 (m, 4H), 4.99–4.88 (m, 1H), 4.44–4.36 (m, 1H), 4.31 (s, 4H), 3.88 (bs, 1H), 3.79–3.65 (m, 1H), 3.58 (bs, 1H), 3.33–2.99 (m, 5H), 2.94–2.51 (m, 7H), 2.35–2.21 (m, 5H), 2.21–2.01 (m, 3H), 1.74–1.40 (m, 3H); ^13^C NMR (126 MHz, CDCl_3_) (mixture of diastereomers): δ, 170.9, 168.5, 168.1, 168.0, 166.9, 166.8, 143.0, 142.6, 142.3, 137.0, 136.1, 135.0, 134.42, 134.37, 134.1, 134.0, 131.82, 131.75, 131.5, 131.4, 131.3, 130.4, 130.2, 129.7, 129.6, 127.3, 127.2, 125.6, 124.9, 124.7, 122.5, 118.5, 118.3, 118.2, 116.9, 115.9, 115.4, 112.8, 100.1, 70.8, 70.6, 70.5, 69.4, 69.3, 66.3, 64.41, 64.39, 62.1, 62.0, 61.25, 61.18, 55.6, 53.8, 53.6, 53.5, 49.1, 44.4, 44.3, 43.6, 40.4, 35.3, 31.1, 29.7, 29.5; HRMS (ESI^+^), m/z calculated for C_57_H_57_O_11_N_7_Cl (M + H)^+^: 1050.3799, found (M + H)^+^: 1050.3796.

(2*R*,4*R*)-1-(5-chloro-2-((3-cyanobenzyl)oxy)-4-((3-(2,3-dihydrobenzo[b][1,4]dioxin-6-yl)-2-methylbenzyl)oxy)benzyl)-N-(3-((2-(2,6-dioxopiperidin-3-yl)-1,3-dioxoisoindolin-4yl)amino)propyl)-4-hydroxypyrrolidine-2-carboxamide (**2**)

HATU (1.2 equiv.) and N,N′-diisopropylethylamine (seven equiv.) were added to a solution of BMS-1166 (0.1 mmol) in 1 mL DMF and stirred. After 10 min, amine-derived pomalidomide hydrochloride salt (PMD-2, 0.1 M in DMSO) was added to the reaction. After 12 h, the reaction mixture was lyophilized (to remove the solvent) and the crude substance was purified by flash column chromatography giving compound **2** as yellow oil with 21% yield. The amine-derived pomalidomide and BMS-1166 used in the synthesis were obtained according to known protocols [28,30].

^1^H NMR (500 MHz, CDCl_3_): δ (ppm), 8.37–8.30 (m, 1H), 7.69 (s, 1H), 7.57 (d, *J* = 7.5 Hz, 2H), 7.56–7.49 (m, 1H), 7.49–7.37 (m, 2H), 7.36–7.26 (m, 1H), 7.25–7.13 (m, 2H), 7.04 (d, *J* = 7.1 Hz, 1H), 6.91 (d, *J* = 8.3 Hz, 1H), 6.83 (d, *J* = 2.0 Hz, 1H), 6.81–6.72 (m, 2H), 6.48 (d, *J* = 1.8 Hz, 1H), 6.33 (t, *J* = 5.4 Hz, 1H), 5.05 (s, 4H), 4.94–4.88 (m, 1H), 4.38 (bs, 1H), 4.32 (s, 4H), 3.89 (d, *J* = 13.1 Hz, 1H), 3.61 (d, *J* = 13.1 Hz, 1H), 3.54 (t, *J* = 8.2 Hz, 1H), 3.28–3.23 (m, 1H), 3.24–3.10 (m, 4H), 2.92–2.84 (m, 1H), 2.82–2.66 (m, 2H), 2.58–2.51 (m, 1H), 2.24 (s, 3H), 2.13–2.05 (m, 2H), 2.04–1.96 (m, 1H), 1.76–1.67 (m, 2H); ^13^C NMR (126 MHz, CDCl_3_): δ (ppm), 174.4, 171.0, 169.3, 168.6, 167.5, 155.2, 154.2, 146.5, 143.0, 142.6, 142.3, 137.9, 136.2, 135.0, 134.4, 133.9, 132.4, 131.8, 131.7, 131.2, 130.3, 130.2, 129.7, 125.5, 122.5, 120.3, 118.5, 118.2, 116.9, 116.4, 115.2, 112.8, 111.5, 110.0, 100.1, 70.6, 70.4, 69.2, 66.1, 64.43, 64.40, 61.3, 54.4, 48.9, 40.4, 39.7, 36.2, 31.4, 29.2, 22.7, 16.2; HRMS (ESI^+^), m/z calculated for C_52_H_50_O_10_N_6_Cl (M + H)^+^: 953.3271, found (M + H)^+^: 953.3267.

2-(4-(((5-chloro-2-((3-cyanobenzyl)oxy)-4-((3-(2,3-dihydrobenzo[b][1,4]dioxin-6-yl)-2-methylbenzyl)oxy)benzyl)amino)methyl)piperidin-1-yl)-N-(2-(2,6-dioxopiperidin-3-yl)-1,3-dioxoisoindolin-4-yl)acetamide (**3**)

Sodium bicarbonate (nine equiv.) was added to a suspension of BMS-1166 aldehyde (0.2 mmol, one equiv.) and amine hydrochloride salt (PMD-1, 0.2 mmol, one equiv.) in dry methanol (1.5 mL). After stirring for 12 h at RT, the mixture was cooled down to 0 °C, and dry dichloroethane (0.5 mL) and sodium triacetoxyborohydride (two equiv. in three portions) were added. After completion of the reaction followed by TLC, the reaction was quenched with water. The solvent was removed in vacuo and purified by flash column chromatography to give the pure product **3** as a yellow solid with 40% yield. The amine-derived pomalidomide and BMS-1166 aldehyde used in the synthesis were obtained according to known protocols [28,30].

^1^H NMR (500 MHz, CDCl_3_): δ, 11.19 (s, 1H), 8/89 (d, *J* = 8.5 Hz, 1H), 7.74 (s, 1H), 7.73–7.66 (m, 1H), 7.61 (dd, *J* = 7.7, 1.7 Hz, 2H), 7.53 (d, *J* = 7.3 Hz, 1H), 7.48 (t, *J* = 7.6 Hz, 1H), 7.44–7.37 (m, 1H), 7.35 (s, 3H), 7.24–7.19 (m, 2H), 6.91 (d, *J* = 8.2 Hz, 1H), 6.81 (d, *J* = 2.1 Hz, 1H), 6.76 (dd, *J* = 8.2, 2.1 Hz, 1H), 5.11 (s, 2H), 5.07 (s, 2H), 4.98–4.91 (m, 1H), 4.30 (s, 4H), 3.77 (s, 2H), 3.16 (s, 2H), 2.93–2.70 (m, 5H), 2.61–2.56 (m, 2H), 2.30–2.23 (m, 5H), 2.27–2.10 (m, 1H), 1.75–1.68 (m, 2H), 1.57–1.47 (m, 3H); ^13^C NMR (126 MHz, CDCl_3_): δ, 171.1, 171.0, 168.3, 168.0, 166.9, 155.3, 153.8, 143.0, 142.6, 142.3, 138.1, 137.1, 136.1, 135.1, 134.6, 134.1, 131.8, 131.1, 130.9, 130.6, 130.2, 129.5, 127.3, 125.5, 125.1, 122.5, 118.4, 118.3, 118.2, 116.9, 116.0, 115.3, 112.9, 100.3, 70.6, 69.3, 64.40, 64.38, 62.4, 55.0, 54.1, 49.1, 48.0, 35.3, 31.3, 30.4, 30.3, 22.6, 16.2; HRMS (ESI^+^), m/z calculated for C_52_H_50_O_9_N_6_Cl (M + H)^+^: 937.3322, found (M + H)^+^: 937.3320.

N-(2-(2,6-dioxopiperidin-3-yl)-1,3-dioxoisoindolin-4-yl)-2-(4-((((2-methoxy-6-((2-methyl-[1,1′-biphenyl]-3-yl)methoxy)pyridin-3-yl)methyl)amino)methyl)piperidin-1-yl)acetamide (**4**)

Sodium bicarbonate (nine equiv.) was added to a suspension of BMS-202 aldehyde (0.2 mmol, one equiv.) and amine hydrochloride salt (PMD-1, 0.2 mmol, one equiv.) in dry methanol (1.5 mL). After stirring for 12 h at RT, the mixture was cooled down to 0 °C, and dry dichloroethane (0.5 mL) and sodium triacetoxyborohydride (two equiv. in three portions) were added. After completion of the reaction followed by TLC, the reaction was quenched with water. The solvent was removed in vacuo and purified by flash column chromatography to give the pure product **4** as a yellow powder with a 34% yield. The amine-derived pomalidomide and BMS-202 aldehyde used in the synthesis were obtained according to known protocols [28,30].

^1^H NMR (500 MHz, CDCl_3_): δ, 11.23 (s, 1H), 8.83 (d, *J* = 8.4 Hz, 1H), 7.77 (d, *J* = 8.1 Hz, 1H), 7.72–7.64 (m, 1H), 7.53 (d, *J* = 7.3 Hz, 1H), 7.43–7.37 (m, 3H), 7.37–7.31 (m, 1H), 7.31–7.27 (m, 2H), 7.25–7.19 (m, 2H), 6.40 (d, *J* = 8.1 Hz, 1H), 5.37 (s, 2H), 5.04–4.89 (m, 1H), 4.06–3.96 (m, 5H), 3.23–3.01 (m, 2H), 2.96–2.59 (m, 7H), 2.43–2.26 (m, 2H), 2.24 (s, 3H), 2.20–2.08 (m, 1H), 1.98 (s, 1H), 1.96–1.72 (m, 3H), 1.67–1.43 (m, 2H); ^13^C NMR (126 MHz, CDCl_3_): δ, 171.6, 170.6, 168.4, 168.2, 166.9, 163.2, 161.0, 143.5, 142.8, 141.9, 136.9, 136.1, 135.3, 134.4, 131.4, 130.1, 129.3, 128.3, 128.0, 126.8, 125.4, 124.9, 118.4, 116.0, 104.9, 102.3, 67.0, 62.0, 53.8, 53.5, 53.3, 51.3, 49.1, 45.2, 32.4, 31.2, 29.9, 29.6, 22.9, 16.2; HRMS (ESI^+^), m/z calculated for C_42_H_45_N_6_O_7_ (M + H)^+^: 745.3344, found (M + H)^+^: 745.3345.

2-(2,6-dioxopiperidin-3-yl)-4-((3-(((2-methoxy-6-((2-methyl-[1,1′-biphenyl]-3-yl)methoxy)pyridin-3-yl)methyl)amino)propyl)amino)isoindoline-1,3-dione (**5**)

Sodium bicarbonate (nine equiv.) was added to a suspension of BMS-202 aldehyde (0.2 mmol, one equiv.) and amine hydrochloride salt (PMD-2, 0.2 mmol, one equiv.) in dry methanol (1.5 mL). After stirring for 12 h at RT, the mixture was cooled down to 0 °C, and dry dichloroethane (0.5 mL) and sodium triacetoxyborohydride (two equiv. in three portions) were added. After completion of the reaction followed by TLC, the reaction was quenched with water. The solvent was removed in vacuo and purified by flash column chromatography to give pure product **5** as a yellow oil with a 73% yield. The amine-derived pomalidomide and BMS-202 aldehyde used in the synthesis were obtained according to known protocols [28,30].

^1^H NMR (500 MHz, CDCl_3_): δ, 8.14 (s, 3H), 7.60 (d, *J* = 8.1 Hz, 1H), 7.46–7.37 (m, 4H), 7.37–7.30 (m, 1H), 7.29 (d, *J* = 7.5 Hz, 2H), 7.24–7.19 (m, 2H), 7.04 (d, *J* = 7.1 Hz, 1H), 6.87 (d, *J* = 8.6 Hz, 1H), 6.39–6.31 (m, 1H), 4.93–4.86 (m, 1H), 3.95 (s, 3H), 3.93 (s, 2H), 3.40–3.33 (m, 2H), 2.95–2.88 (m, 2H), 2.85–2.64 (m, 4H), 2.24 (s, 3H), 2.10–1.99 (m, 3H), 1.98 (s, 3H); ^13^C NMR (126 MHz, CDCl_3_): δ, 171.6, 169.4, 168.8, 167.6, 162.8, 160.8, 146.6, 142.9, 142.7, 142.0, 136.2, 135.6, 134.5, 132.5, 130.1, 129.4, 128.4, 128.1, 126.8, 125.5, 116.7, 111.7, 110.3, 101.9, 67.0, 53.6, 48.9, 46.1, 44.5, 40.1, 31.4, 27.1, 22.8, 16.3; HRMS (ESI^+^), m/z calculated for C_37_H_38_O_6_N_5_ (M + H)^+^: 648.2817, found (M + H)^+^: 648.2817.

3-((2-((1-(*tert*-butyl)-1*H*-tetrazol-5-yl)((3-((2-(2,6-dioxopiperidin-3-yl)-1,3-dioxoisoindolin-4-yl)amino)propyl)amino)methyl)-4-chloro-5-((3-(2,3-dihydrobenzo[b][1,4]dioxin-6-yl)-2-methylbenzyl)oxy)phenoxy)methyl)benzonitrile (**6**)

To the stirred solution of the BMS-1166 aldehyde component (0.2 mmol, 1.0 equiv.) and amine hydrochloride salt (PMD-2, 0.2 mmol, 1.0 equiv.) in methanol/water/dichloromethane (1.6 mL, 1:0.3:0.3), *tert*-butyl isocyanide (1.2 equiv.) and sodium azide (1.5 equiv.) were added at 0 °C. After addition, the reaction was stirred at room temperature for 18 h. The reaction was concentrated in vacuo and purified by flash column chromatography to give pure product **6** as a yellow oil with a 20% yield. The amine-derived pomalidomide and BMS-1166 aldehyde used in the synthesis were obtained according to known protocols [28,30].

^1^H NMR (500 MHz, CDCl_3_) (mixture of diastereomers): δ, 8.10–7.94 (m, 1H), 7.69 (s, 1H), 7.65–7.59 (m, 1H), 7.56–7.49 (m, 2H), 7.42–7.35 (m, 1H), 7.26–7.21 (m, 3H), 7.06 (t, *J* = 6.9 Hz, 1H), 6.91 (d, *J* = 8.2 Hz, 1H), 6.84–6.79 (m, 2H), 6.77 (dd, *J* = 8.2, 2.1 Hz, 1H), 6.61 (d, *J* = 3.8 Hz, 1H), 6.56–6.44 (m, 1H), 5.71 (d, *J* = 4.0 Hz, 1H), 5.19–5.05 (m, 4H), 4.91 (m, 1H), 4.31 (s, 4H), 3.39–3.23 (m, 2H), 2.91–2.80 (m, 1H), 2.79–2.67 (m, 3H), 2.67–2.58 (m, 1H), 2.27–2.23 (m, 3H), 2.15–2.02 (m, 1H), 1.84–1.75 (m, 2H), 1.59 (s, 9H); ^13^C NMR (126 MHz, CDCl_3_) (mixture of diastereomers): δ, 172.2, 170.9, 168.4, 167.6, 155.1, 154.3, 146.6, 143.0, 142.7, 142.4, 137.7, 136.1, 135.0, 134.2, 132.4, 132.0, 131.4, 130.6, 130.3, 127.3, 125.6, 122.5, 121.0, 118.2, 116.9, 112.9, 111.4, 109.8, 100.2, 70.6, 69.8, 64.42, 64.40, 61.2, 50.9, 48.7, 41.1, 31.4, 29.7, 29.6, 29.0, 22.7, 16.3; HRMS (ESI^+^), m/z calculated for C_52_H_51_O_8_N_9_Cl (M + H)^+^: 964.3544, found (M +H)^+^: 964.3542.

4-((3-(((1-(*tert*-butyl)-1*H*-tetrazol-5-yl)(2-methoxy-6-((2-methyl-[1,1′-biphenyl]-3-yl)methoxy)pyridin-3-yl)methyl)amino)propyl)amino)-2-(2,6-dioxopiperidin-3-yl)isoindoline-1,3-dione (**7**)

To the stirred solution of the BMS-202 aldehyde component (0.2 mmol, 1.0 equiv.) and amine hydrochloride salt (PMD-2, 0.2 mmol, 1.0 equiv.) in methanol/water/dichloromethane (1.6 mL, 1:0.3:0.3), *tert*-butyl isocyanide (1.2 equiv.) and sodium azide (1.5 equiv.) were added at 0 °C. After addition, the reaction was stirred at room temperature for 18 h. The reaction was concentrated in vacuo and purified by flash column chromatography to give pure product **7** as a yellow oil with a 78% yield. The amine-derived pomalidomide and BMS-202 aldehyde used in the synthesis were obtained according to known protocols [28,30].

^1^H NMR (500 MHz, CDCl_3_) (mixture of diastereomers): δ, 8.15 (d, *J* = 8.1 Hz, 1H), 7.55 (dd, *J* = 8.2, 14. Hz, 1H), 7.49–7.43 (m, 1H), 7.43–7.38 (m, 3H), 7.37–7.33 (m, 1H), 7.32–7.28 (m, 2H), 7.26–7.21 (m, 2H), 7.07 (d, *J* = 7.1 Hz, 1H), 6.88 (d, *J* = 7.1 Hz, 1H), 6.51–6.46 (m, 1H), 6.38 (d, *J* = 8.2 Hz, 1H), 5.67 (d, *J* = 3.1 Hz, 1H), 5.47–5.35 (m, 2H), 4.92–4.84 (m, 1H), 4.01 (s, 3H), 3.45–3.29 (m, 2H), 2.92–2.63 (m, 5H), 2.26 (s, 3H), 2.20–2.06 (m, 1H), 1.87–1.76 (m, 2H), 1.66 (s, 9H); ^13^C NMR (126 MHz, CDCl_3_) (mixture of diastereomers): δ (ppm), 171.1, 170.9, 169.3, 168.5, 167.6, 162.3, 159.3, 155.6, 146.9, 142.9, 142.0, 140.1, 136.1, 135.5, 134.5, 132.5, 130.1, 129.4, 128.3, 128.1, 126.9, 125.5, 116.6, 112.2, 111.4, 110.0, 102.6, 67.0, 61.3, 53.8, 50.6, 48.9, 45.2, 40.9, 31.4, 29.8, 29.4, 22.8, 16.3; HRMS (ESI^+^), m/z calculated for C_42_H_46_O_6_N_9_ (M + H)^+^: 772.3566, found (M + H)^+^: 772.3565.

4-((2′-chloro-3′-(2,3-dihydrobenzo[b][1,4]dioxin-6-yl)-3-methoxy-[1,1′-biphenyl]-4-yl)methoxy)-4-oxobutanoic acid (**8**)

DCM (1.5 mL), m-terphenyl core (0.50 mmol, 1.0 equiv.), succinic anhydride (1.05 equiv.), diisopropylethylamine (1.7 equiv.), and DMAP (0.1 equiv.) were added to a round-bottom flask. The reaction mixture was stirred for 24 h at room temperature in the argon atmosphere. After this time, the solvent was evaporated, and the resulting slurry was dissolved in CHCl_3_ and washed twice with 1M HCl. Then, the organic layer was dried over anhydrous MgSO_4_ and evaporated to obtain product **8** with a 94% yield with sufficient purity. The m-terphenyl core used in the synthesis was obtained according to a known protocol [31].

^1^H NMR (600 MHz, CDCl_3_): δ (ppm), 7.37 (d, *J* = 7.64 Hz, 1H), 7.34–7.30 (m, 2H), 7.29 (dd, *J* = 2.64; 6.71 Hz, 1H), 7.03 (dd, *J* = 1.41; 7.64 Hz, 1H), 7.00 (dd, *J* = 1.56; 6.41, 2H), 6.95 (dd, *J* = 2.04; 8.27, 1H), 6.93 (d, *J* = 8.27 Hz, 1H), 5.25 (s, 2H), 4.31 (s, 4H), 3.86 (s, 3H), 2.73 (s, 4H); ^13^C NMR (151 MHz, CDCl_3_): δ (ppm), 176.1, 172.2, 157.0, 143.3, 143.0, 141.7, 141.3, 141.1, 133.3, 130.9, 130.6, 130.1, 129.3, 126.4, 123.2, 122.8, 121.6, 118.6, 116.9, 112.1, 64.5, 64.4, 62.1, 55.6, 29.0, 28.7; HRMS (ESI) calc. for C_26_H_23_ClO_7_ (m/z): (M + Na)^+^, 505.1025; found: (M + Na)^+^, 505.1025.

4-((3′-(benzo-1,4-dioxan-6-yl)-2′-chloro-3-methoxy-[1,1′-biphenyl]-4-yl)methoxy)-4-oxopentanoic acid (**9**)

DCM (2.5 mL), m-terphenyl core (0.86 mmol, 1.0 equiv.), glutaric anhydride (1.05 equiv.), diisopropylethylamine (1.3 equiv.), and DMAP (0.05 equiv.) were added to a round-bottom flask. The reaction mixture was stirred for 24 h at room temperature in the argon atmosphere. After this time, the solvent was evaporated, and the resulting slurry was dissolved in CHCl_3_ and washed twice with 1M HCl. Then, the organic layer was dried over anhydrous MgSO_4_ and evaporated. The crude product was purified using column chromatography (hexane to hexane/AcOEt 1:1) to obtain product **9** with a 62% yield. The m-terphenyl core used in the synthesis was obtained according to a known protocol [31].

^1^H NMR (600 MHz, CDCl_3_): δ (ppm), 12.13 (s, 1H), 7.44 (t, *J* = 7.5 Hz, 1H), 7.38 (dd, *J* = 7.6, 1.8 Hz, 1H), 7.37–7.35 (m, 2H), 7.08 (d, *J* = 1.4 Hz, 1H), 7.01 (dd, *J* = 7.7, 1.5 Hz, 1H), 6.95–6.92 (m, 2H), 6.90 (dd, *J* = 8.3, 2.0 Hz, 1H), 5.11 (s, 2H), 4.28 (s, 4H), 3.83 (s, 3H), 2.41 (t, *J* = 7.4 Hz, 2H), 2.27 (t, *J* = 7.4 Hz, 2H), 1.77 (quin, *J* = 7.4 Hz, 2H); ^13^C NMR (151 MHz, CDCl_3_): δ (ppm), 174.5, 173.0, 157.1, 143.6, 143.3, 141.4, 141.2, 140.9, 132.9, 131.1, 130.7, 130.3, 129.5, 127.5, 123.7, 122.8, 121.8, 118.5, 117.2, 112.7, 64.6, 61.3, 56.1, 33.1, 33.1, 20.5; HRMS (ESI) calc. for C_27_H_25_ClO_7_ (m/z): (M + Na)^+^, 519.1181; found: (M + Na)^+^, 519.1182.

(3′-(benzo-1,4-dioxan-6-yl)-2′-chloro-3-methoxy-[1,1′-biphenyl]-4-yl)methyl 4-(4-(2-(2,6-dioxopiperidin-3-yl)-1,3-dioxoisoindolin-4-yl)piperazin-1-yl)-4-oxobutanoate (**10**)

Compound **8** (0.22 mmol, one equiv.) and DMF (2 mL) were added to a round-bottom flask. The reaction mixture was charged with the argon atmosphere, and then diisopropylethylamine (three equiv.), DMF (10 drops), and HATU (1.1 equiv.) were added. Then, the reaction was stirred for 30 min at room temperature; then, amine-derived pomalidomide (PMD-3, 0.104 g as a TFA salt, one equiv.) in DMF (2 mL) was added and stirred for 30 min at room temperature. The mixture was diluted in AcOEt (20 mL) and washed with water (10 mL) and brine (3 × 10 mL). The organic layer was dried over anhydrous MgSO_4_ and evaporated. The crude product was purified on a column (hexane to AcOEt) to obtain final product **10** with a 40% yield. The amine-derived pomalidomide used in the synthesis was obtained according to a known protocol [37].

^1^H NMR (600 MHz, CDCl_3_): δ (ppm), 11.09 (s, 1H), 7.71 (dd, *J* = 7.3, 8.3 Hz; 1H), 7.43 (t, *J* = 7.5 Hz, 1H), 7.40–7.33 (m, 5H), 7.08 (d, *J* = 1.3 Hz, 1H), 7.01 (dd, *J* = 5.1, 3.1 Hz), 6.95–6.92 (m, 2H), 6.90–6.88 (m, 1H), 5.13–5.09 (m, 3H), 4.29 (s, 4H), 3.84 (s, 3H), 3.69–3.62 (m, 4H), 3.31 (s_broad_, 2H), 3.24 (s_broad_, 2H), 2.92–2.83 (m, 1H), 2.71–2.67 (m, 6H), 2.64–2.60 (m, 2H), 2.05–2.00 (m, 1H); ^13^C NMR (151 MHz, CDCl_3_): δ (ppm), 172.8, 172.4, 172.0, 169.6, 167.0, 166.4, 156.5, 149.4, 143.1, 142.9, 140.8, 140.5, 135.9, 133.6, 132.4, 130.6, 130.3, 129.9, 128.7, 127.0, 123.9, 123.3, 122.4, 121.3, 118.0, 116.9, 116.7, 116.7, 115.2, 112.2, 64.1, 64.1, 60.7, 55.7, 50.8, 50.2, 48.8, 44.7, 41.2, 38.2, 30.9, 28.9, 27.4, 22.0; HRMS (ESI) calc. for C_43_H_39_ClN_4_O_10_ (m/z): (M + Na)^+^, 829.2246; found: (M + H)^+^, 829.2246.

(3′-(benzo-1,4-dioxan-6-yl)-2′-chloro-3-methoxy-[1,1′-biphenyl]-4-yl)methyl 4-(4-(2-(2,6-dioxopiperidin-3-yl)-1,3-dioxoisoindolin-4-yl)piperazin-1-yl)-4-oxopentanoate (**11**)

Compound **9** (0.27 mmol, 1 equiv.) and DMF (2 mL) were added to a round-bottom flask. The reaction mixture was charged with the argon atmosphere, and then diisopropylethylamine (3 equiv.), DMF (10 drops) and HATU (1.1 equiv.) were added. Then, the reaction was stirred for 30 min at room temperature; then, amine-derived pomalidomide (PMD-3, 0.102 g as a TFA salt, one equiv.) in DMF (2 mL) was added and stirred for 30 min at room temperature. The mixture was diluted in AcOEt (20 mL) and washed with water (10 mL) and brine (3 × 10 mL). The organic layer was dried over anhydrous MgSO_4_ and evaporated. The crude product was purified on a column (hexane to AcOEt) to obtain final product **11** with a 79% yield. The amine-derived pomalidomide used in the synthesis was obtained according to a known protocol [37].

^1^H NMR (600 MHz, CDCl_3_): δ (ppm), 12.13 (s, 1H), 7.44 (t, *J* = 7.5 Hz, 1H), 7.38 (dd, *J* = 7.6, 1.8 Hz, 1H), 7.37–7.35 (m, 2H), 7.08 (d, *J* = 1.4 Hz, 1H), 7.01 (dd, *J* = 7.7, 1.5 Hz, 1H), 6.95–6.92 (m, 2H), 6.90 (dd, *J* = 8.3, 2.0 Hz, 1H), 5.11 (s, 2H), 4.28 (s, 4H), 3.03 (s, 3H), 2.41 (t, *J* = 7.4 Hz, 2H), 2.27 (t, *J* = 7.4 Hz, 2H), 2.03 (quin., *J* = 7.4 Hz, 2H); ^13^C NMR (151 MHz, CDCl_3_) δ (ppm), 174.5, 173.0, 157.1, 143.6, 143.3, 141.4, 141.2, 140.9, 132.9, 131.1, 130.7, 130.3, 129.5, 127.5, 123.7, 122.8, 121.8, 118.5, 117.2, 112.7, 64.6, 61.3, 56.1, 33.11, 33.07, 20.5 LC–MS (DAD/ESI): t_r_ 7.89 = min, calc. for C_44_H_41_ClN_4_O_10_ (m/z): (M + H)^+^, 821.26; found: (M + H)^+^, 821.46.

N1-((3-((3-cyanobenzyl)amino)-6-((4′-fluoro-2-methyl-[1,1′-biphenyl]-3-yl)methoxy)imidazo [1,2-a]pyridin-2-yl)methyl)-N5-(2-(2,6-dioxopiperidin-3-yl)-1,3-dioxoisoindolin-4-yl)glutaramide (**12**)

HATU (1.2 equiv.) and N,N′-diisopropylethylamine (7.0 equiv.) were added to a solution of carboxylic acid-derived pomalidomide (PMD-4, 0.1 mmol) in 1 mL DMF and stirred. After 10 min, imidazopyridine amine hydrochloride salt (0.1 M in DMSO) was added to the reaction. After 12 h, the reaction mixture was lyophilized (to remove the solvent) and the crude substance was purified by flash column chromatography giving product **12** as a yellow powder with a 43% yield. The carboxylic acid-derived pomalidomide and imidazopyridine aldehyde used in the synthesis were obtained according to known protocols [24,28].

^1^H NMR (500 MHz, CDCl_3_): δ, 11.47 (s, 1H), 8.66 (d, *J* = 8.3 Hz, 1H), 7.74–7.67 (m, 1H), 7.66 (s, 1H), 7.62 (d, *J* = 9.7 Hz, 1H), 7.59–7.54 (m, 1H), 7.57–7.51 (m, 3H), 7.43–7.37 (m, 2H), 7.29–7.20 (m, 4H), 7.15–7.07 (m, 2H), 7.05 (t, *J* = 6.2 Hz, 1H), 7.01 (dd, *J* = 9.7, 2.4 Hz, 1H), 5.05 (t, *J* = 6.9 Hz, 1H), 4.97 (s, 2H), 4.97–4.92 (m, 1H), 4.15 (d, *J* = 5.8 Hz, 4H), 2.93–2.84 (m, 1H), 2.82–2.63 (m, 2H), 2.53–2.37 (m, 2H), 2.31–2.15 (m, 6H), 2.12–1.92 (m, 1H); ^13^C NMR (126 MHz, CDCl_3_): δ, 172.7, 172.1, 171.6, 169.4, 166.7, 163.0, 161.0, 148.3, 142.1, 141.1, 138.5, 137.59, 137.57, 137.46, 136.4, 134.6, 134.42, 134.38, 132.8, 131.8, 131.2, 131.1, 130.9, 130.8, 130.6, 129.4, 128.4, 127.8, 125.7, 119.7, 118.7, 118.6, 117.8, 115.8, 115.1, 115.0, 112.6, 105.4, 70.3, 51.8, 49.5, 36.8, 35.7, 35.5, 31.5, 23.1, 21.2, 16.2; HRMS (ESI^+^), m/z calculated for C_48_H_42_O_7_N_8_F [M + H]^+^: 861.3155, found [M + H]^+^: 861.3162.

N1-(2-(2,6-dioxopiperidin-3-yl)-1,3-dioxoisoindolin-4-yl)-N5-((6-((2-methyl-[1,1′-biphenyl]-3-yl)methoxy)-3-((1-phenylethyl)amino)imidazo [1,2-a]pyridin-2-yl)methyl)glutaramide (**13**)

HATU (1.2 equiv.) and N,N′-diisopropylethylamine (7.0 equiv.) were added to a solution of carboxylic acid-derived pomalidomide (PMD-4, 0.1 mmol) in 1 mL DMF and stirred. After 10 min, amine hydrochloride salt (0.1 M in DMSO) was added to the reaction. After 12 h, the reaction mixture was lyophilized (to remove the solvent) and the crude substance was purified by flash column chromatography giving product **13** as a yellow oil with a 25% yield. The carboxylic acid-derived pomalidomide and imidazopyridine aldehyde used in the synthesis were obtained according to known protocols [24,28].

^1^H NMR (500 MHz, CDCl_3_) (mixture of diastereomers): δ, 9.38 (s, 1H), 8.67 (d, *J* = 8.4 Hz, 1H), 7.74–7.67 (m, 1H), 7.61–7.51 (m, 2H), 7.48–7.26 (m, 12H), 7.26–7.18 (m, 1H), 6.99–6.89 (m, 2H), 4.97–4.70 (m, 4H), 4.37–4.07 (m, 3H), 2.92–2.84 (m, 1H), 2.80–2.62 (m, 2H), 2.52–2.39 (m, 2H), 2.28–2.15 (m, 4H), 2.13–1.96 (m, 4H), 1.65–1.59 (m, 3H); ^13^C NMR (126 MHz, CDCl_3_) (mixture of diastereomers): δ, 172.40, 172.36, 171.75, 171.73, 171.67, 171.65, 169.3, 169.18, 169.15, 147.9, 147.8, 144.8, 144.6, 143.12, 143.09, 141.7, 138.39, 138.36, 137.5, 136.4, 134.48, 134.46, 134.42, 131.1, 130.5, 129.3, 128.6, 128.5, 128.3, 128.2, 128.1, 128.0, 127.4, 126.9, 126.8, 126.7, 125.9, 125.6, 119.54, 119.48, 118.7, 117.31, 117.26, 115.9, 105.9, 105.8, 70.1, 70.0, 58.6, 58.1, 49.5, 36.9, 35.8, 35.6, 35.39, 35.36, 31.4, 23.1, 23.0, 22.8, 21.3, 16.20, 16.19; HRMS (ESI^+^), m/z calculated for C_48_H_46_O_7_N_7_ (M + H)^+^: 832.3453, found (M + H)^+^, 832.3465.

### 4.2. Protein Expression and Purification

The *E. coli* strain BL21 was transformed with a pET-21b plasmid carrying the PD-L1 gene (amino acids 18–239). The bacteria were cultured in LB at 37 °C until OD_600_nm of 0, 6 when the recombinant protein production was induced with 1 mM IPTG. The protein production continued overnight. The inclusion bodies were collected by centrifugation, washed twice with 50 mM Tris HCl, pH 8.0, containing 200 mM NaCl, 10 mM EDTA, 10 mM 2-mercaptoethanol, and 0.5% Triton X-100, followed by a single wash with the same buffer but with no Triton X-100. The washed inclusion bodies were resuspended overnight in 50 mM Tris HCl, pH 8.0, 6 M GuHCl, 200 mM NaCl, and 10 mM 2-mercaptoethanol and clarified with centrifugation. Refolding of PD-L1 was performed by dropwise dilution into 0.1 M Tris HCl, pH 8.0, containing 1 M L-arginine hydrochloride, 0.25 mM oxidized glutathione, and 0.25 mM reduced glutathione. The refolded protein was dialyzed three times against 10 mM Tris HCl, pH 8.0, containing 20 mM NaCl and purified by size exclusion chromatography using Superdex 75 and a dialysis buffer. The quality of the refolded protein was evaluated by SDS-PAGE and NMR.

### 4.3. Homogenous Time-Resolved Fluorescence

HTRF was carried out using a certified Cis-Bio assay according to the manufacturer’s guidelines. The experiments were carried out at 5 nM of h-L1 and 50 nM of hPD-1 in the final formulation at the 20 µL final volume in the well. To determine the half maximal inhibitory concentrations (IC_50_) of the tested compounds, the measurements were carried out on two individual dilution series unless stated otherwise. After mixing all the components according to the Cis-Bio protocol, the plate was left for 2 h incubation at room temperature followed by the TR-FRET measurement on a Tecan Spark 20M. Inhibitor activities were first evaluated by their activity at two different concentrations (5 µM and 0.5 µM concentrations of the tested inhibitor). The best scoring compounds were evaluated with full IC_50_ determination at six different ligand concentrations. The collected data was background-subtracted from the negative control, normalized on the positive control, averaged, and fitted with the normalized Hill’s equation to determine the IC_50_ value using *Mathematica 12* (Princeton, NJ, USA).

### 4.4. NMR Binding Analysis

NMR spectra were recorded in PBS, pH 7.4, containing 10% (*v*/*v*) of D_2_O added to the samples to provide the lock signal. Water suppression was carried out using the WATERGATE sequence. All the spectra were recorded at 300 K using a Bruker Avance 600 MHz spectrometer with the Cryo-Platform. The NMR spectrum was recorded at three different ligand/protein ratios from 0 to 10. The samples were prepared by adding small amounts of a 50 mM ligand stock solution in DMSO to the protein solution (0.20 mL) of the PD-L1 fragment at a concentration of 0.14 mM. The acquisition parameters for each spectrum were as follows: size of FID32768, number of scans: 32. The spectra were visualized using *TopSpin 4.0.2.* (Bruker, Billerica, MA, USA)

### 4.5. Cell Culture

The human PD-L1-expressing CHO-K1 cells overexpressing TCR activator (CHO/TCRAct/PD-L1) cells and Jurkat ECs T cells overexpressing PD-1 and carrying a luciferase reporter gene under the control of the Nuclear Factor of Activated T cells Response Element (NFAT-RE) were obtained from Promega and cultured in an RPMI-1640 medium (Biowest, Nuaillé, France) supplemented with 10% fetal bovine serum (FBS, Biowest, Nuaillé, France) and 200 mM L-glutamine (Biowest, Nuaillé, France). G418 (250 µg/mL, InvivoGen, San Diego, CA, USA) and Hygromycin B Gold (50 µg/mL, InvivoGen, San Diego, CA, USA) were also added to the culture medium as selection antibiotics. CAKI 2, LNCap, PC-3, and MDA-MB-231 cells were purchased from the European Collection of Authenticated Cell Cultures (ECACC) and cultured according to the provided protocol.

### 4.6. Immune Checkpoint Blockade (ICB) Assay

For the analysis of biological activity of the analyzed compounds, an immune checkpoint blockade (ICB) assay was performed. For this, the culture of Jurkat effector cells (Jurkat-ECs) and CHO/TCR-Act/PD-1 was carried out. The assay was performed according to the protocol described in our previous works [18]. All the experiments were performed three times as independent experiments.

### 4.7. Western Blotting

The cell lysates were prepared with a RIPA buffer (Sigma-Aldrich) containing Protease Inhibitor Cocktail (Sigma-Aldrich, St. Louis, MO, USA) and separated by 12% SDS-PAGE (TGX™ FastCast™ Acrylamide Kit, Bio-Rad, Hercules, CA, USA). The proteins were transferred to a PVDF membrane (Merck Millipore, Burlington, MA, USA) using a Mini Trans-Blot^®^ Cell system (Bio-Rad, Hercules, CA, USA). The membranes were incubated with 4% (*m*/*v*) bovine serum albumin (BioShop, Burlington, Ontario, Canada) in a TBS buffer containing 0.1% (*v*/*v*) Nonidet P-40 (BioShop, Burlington, Ontario, Canada) at RT for 1 h. Then, the membranes were incubated with a specific primary antibody at 4 °C overnight. Following three washes in TBS-N, a secondary horseradish peroxidase (HRP)-conjugated antibody was applied for 1 h at RT. After three additional washes in TBS-N, the visualization of the protein with Clarity Western ECL Substrate (Bio-Rad, Hercules, CA, USA) and ChemiDoc MP Imaging System (Bio-Rad, Hercules, CA, USA) was carried out according to the manufacturer’s instructions. The following antibodies were used: rabbit monoclonal anti-PD-L1 antibody at a 1:1000 dilution (clone E1L3N, Cell Signaling Technology, CST, cat. #13684), rabbit monoclonal anti-GAPDH at 1:2000 (clone 14C10, CST, cat. #2118), and goat HRP-linked anti-rabbit secondary antibody at 1:3000 (CST, cat. #7074).

## Figures and Tables

**Table 1:** Activities and structure of the tested chimeras. Red: PD-1/PD-L1 inhibitor, black: linker, blue: pomalidomide.

No.	Structure	HTRF: Percentage of the Dissociated PD-1/PD-L1 Complex			Promega Assay
		5 µM Ligand Conc.	0.5 µM Ligand Conc.	IC_50_ (µM)	
**1**	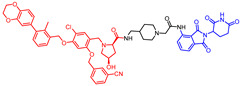	68.3	23.7	-	Not active
**2**	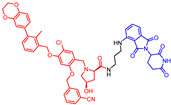	71.7	3.9	-	Not active
**3**	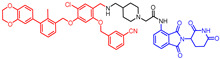	97.1	77.8	0.06 ± 0.002	Not active
**4**	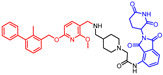	88.8	18.2	1.32 ± 0.04	Active
**5**	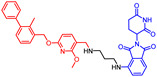	100.0	66.3	0.64 ± 0.02	Active
**6**	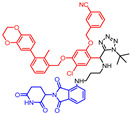	25.9	13.8	-	Not active
**7**	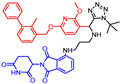	35.2	0.0	-	Not active
**10**	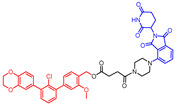	60.6	29.4	-	Not active
**11**	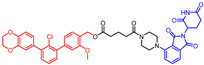	40.0	26.3	-	Not active
**12**	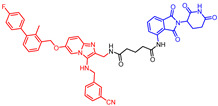	76.3	40.9	-	Not active
**13**	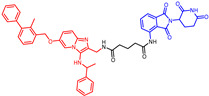	31.1	30.4	-	Not active

## Data Availability

The datasets generated and analyzed during the current study are included in this published article (and its Supplementary Information files) or available from the corresponding author on reasonable request.

## Supplementary Materials

The following supporting information can be downloaded at: https://www.mdpi.com/article/10.3390/molecules27113454/s1: Figure S1: The aliphatic 1H NMR spectrum of the reference PD-L1 (blue) superimposed on the spectrum of the PD-L1 titrated with pomalidomide. Figure S2: The bioactivity of the Pomalidomide as monitored with a cell-based immune checkpoint blockade (ICB) assay. Copies of the NMR spectra of final compounds. Figure S3: (2R,4R)-1-(5-chloro-2-((3-cyanobenzyl)oxy)-4-((3-(2,3-dihydrobenzo[b][1,4]dioxin-6-yl)-2-methylbenzyl)oxy)benzyl)-N-((1-(2-((2-(2,6-dioxopiperidin-3-yl)-1,3-dioxoisoindolin-4-yl)amino)-2-oxoethyl)piperidin-4-yl)methyl)-4-hydroxypyrrolidine-2-carboxamide (**1**). Figure S4: (2R,4R)-1-(5-chloro-2-((3-cyanobenzyl)oxy)-4-((3-(2,3-dihydrobenzo[b][1,4]dioxin-6-yl)-2-methylbenzyl)oxy)benzyl)-N-(3-((2-(2,6-dioxopiperidin-3-yl)-1,3-dioxoisoindolin-4-yl)amino)propyl)-4-hydroxypyrrolidine-2-carboxamide (**2**). Figure S5: 2-(4-(((5-chloro-2-((3-cyanobenzyl)oxy)-4-((3-(2,3-dihydrobenzo[b][1,4]dioxin-6-yl)-2-methylbenzyl)oxy)benzyl)amino)methyl)piperidin-1-yl)-N-(2-(2,6-dioxopiperidin-3-yl)-1,3-dioxoisoindolin-4-yl)acetamide (**3**). Figure S6: N-(2-(2,6-dioxopiperidin-3-yl)-1,3-dioxoisoindolin-4-yl)-2-(4-((((2-methoxy-6-((2-methyl-[1,1′-biphenyl]-3-yl)methoxy)pyridin-3-yl)methyl)amino)methyl)piperidin-1-yl)acetamide (**4**). Figure S7: 2-(2,6-dioxopiperidin-3-yl)-4-((3-(((2-methoxy-6-((2-methyl-[1,1′-biphenyl]-3-yl)methoxy)pyridin-3-yl)methyl)amino)propyl)amino)isoindoline-1,3-dione (**5**). Figure S8: 2-(2,6-dioxopiperidin-3-yl)-4-((3-(((2-methoxy-6-((2-methyl-[1,1′-biphenyl]-3-yl)methoxy)pyridin-3-yl)methyl)amino)propyl)amino)isoindoline-1,3-dione (**5**). Figure S9: 3-((2-((1-(*tert*-butyl)-1H-tetrazol-5-yl)((3-((2-(2,6-dioxopiperidin-3-yl)-1,3-dioxoisoindolin-4-yl)amino)propyl)amino)methyl)-4-chloro-5-((3-(2,3-dihydrobenzo[b][1,4]dioxin-6-yl)-2-methylbenzyl)oxy)phenoxy)methyl)benzonitrile (**6**). Figure S10: 4-((3-(((1-(*tert*-butyl)-1H-tetrazol-5-yl)(2-methoxy-6-((2-methyl-[1,1′-biphenyl]-3-yl)methoxy)pyridin-3-yl)methyl)amino)propyl)amino)-2-(2,6-dioxopiperidin-3-yl)isoindoline-1,3-dione (**7**). Figure S11: (3′-(benzo-1,4-dioxan-6-yl)-2′-chloro-3-methoxy-[1,1′-biphenyl]-4-yl)methyl 4-(4-(2-(2,6-dioxopiperidin-3-yl)-1,3-dioxoisoindolin-4-yl)piperazin-1-yl)-4-oxobutanoate (**10**). Figure S12: (3′-(benzo-1,4-dioxan-6-yl)-2′-chloro-3-methoxy-[1,1′-biphenyl]-4-yl)methyl 4-(4-(2-(2,6-dioxopiperidin-3-yl)-1,3-dioxoisoindolin-4-yl)piperazin-1-yl)-4-oxopentanoate (**11**). Figure S13: N1-((3-((3-cyanobenzyl)amino)-6-((4′-fluoro-2-methyl-[1,1′-biphenyl]-3-yl)methoxy)imidazo[1,2-a]pyridin-2-yl)methyl)-N5-(2-(2,6-dioxopiperidin-3-yl)-1,3-dioxoisoindolin-4-yl)glutaramide (**12**). Figure S14: N1-(2-(2,6-dioxopiperidin-3-yl)-1,3-dioxoisoindolin-4-yl)-N5-((6-((2-methyl-[1,1′-biphenyl]-3-yl)methoxy)-3-((1-phenylethyl)amino)imidazo[1,2-a]pyridin-2-yl)methyl)glutaramide (**13**).

## Sample Availability

Samples of the compounds are available from the authors.
