# Supplementary material for: Biphenyl Ether Analogs Containing Pomalidomide as Small-Molecule Inhibitors of the Programmed Cell Death-1/Programmed Cell Death-Ligand 1 Interaction
Source: Molecules. 2022 May 27;27(11):3454. doi: 10.3390/molecules27113454 (PMC9181982; doi:10.3390/molecules27113454)
Supplement: Supplementary file 1 [file molecules-27-03454-s001.zip › molecules-1706384-supplementary.pdf]

Supplementary Materials

Biphenyl Ether Analogs Containing Pomalidomide as Small-Molecule Inhibitors of the Programmed Cell Death-1/Programmed Cell Death-Ligand 1 Interaction

Copies of the NMR spectra of final compounds

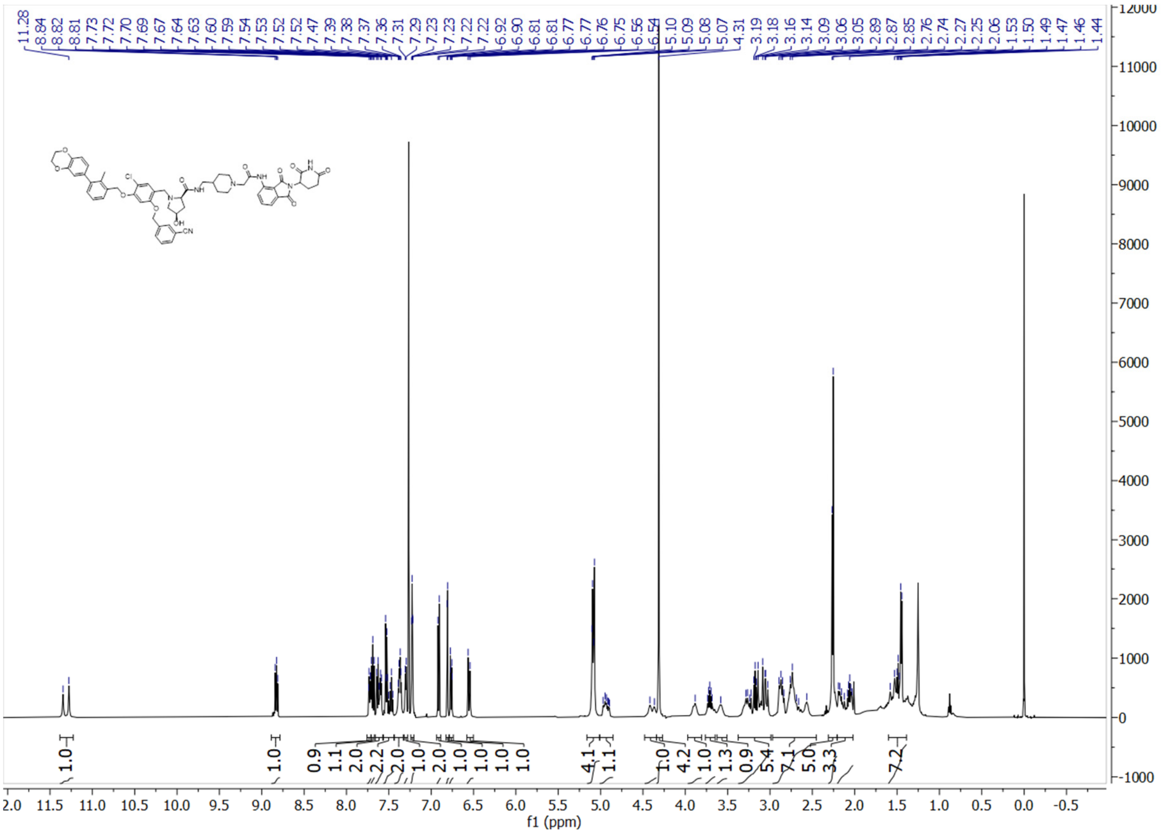

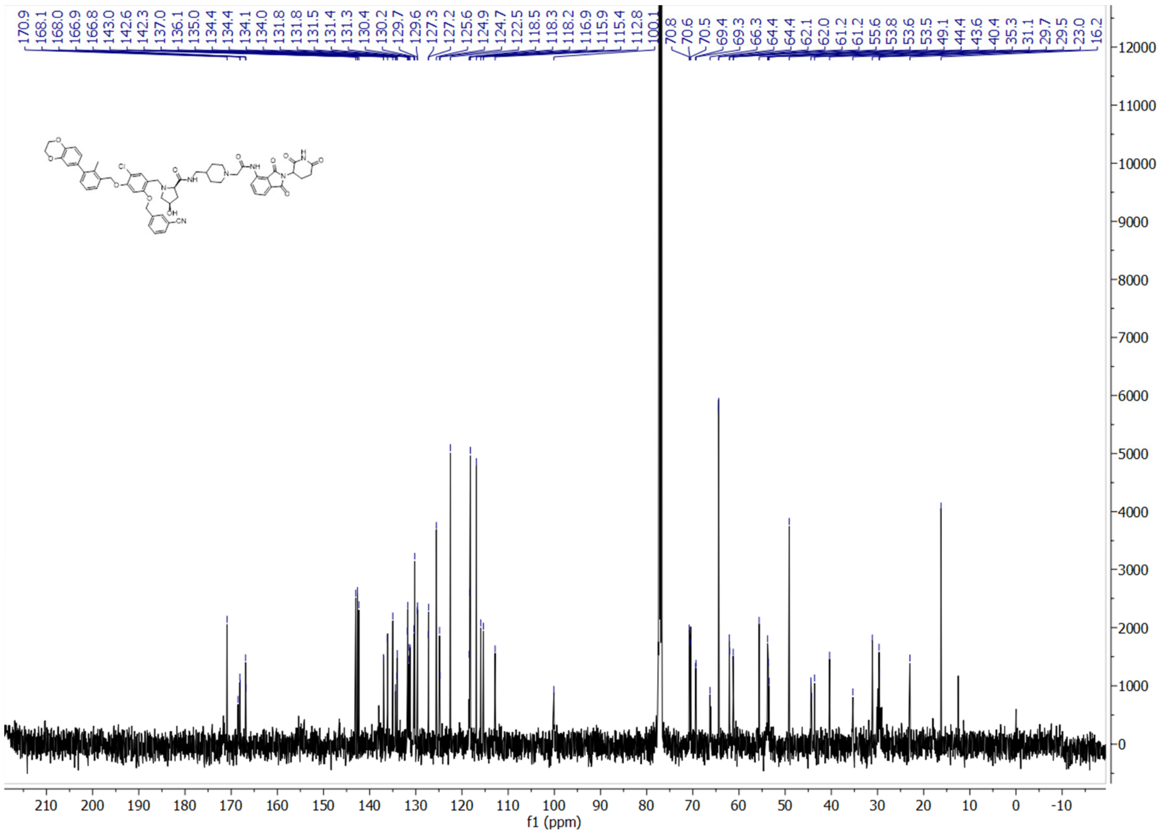

Figure S1. (2R,4R)-1-(5-chloro-2-((3-cyanobenzyl)oxy)-4-((3-(2,3-dihydrobenzo[b][1,4]dioxin-6-yl)-2-methylbenzyl)oxy)benzyl)-N-((1-(2-((2-(2,6-dioxopiperidin-3-yl)-1,3-dioxoisoindolin-4-yl)amino)-2-oxoethyl)piperidin-4-yl)methyl)-4-hydroxypyrrolidine-2-carboxamide (**1**).

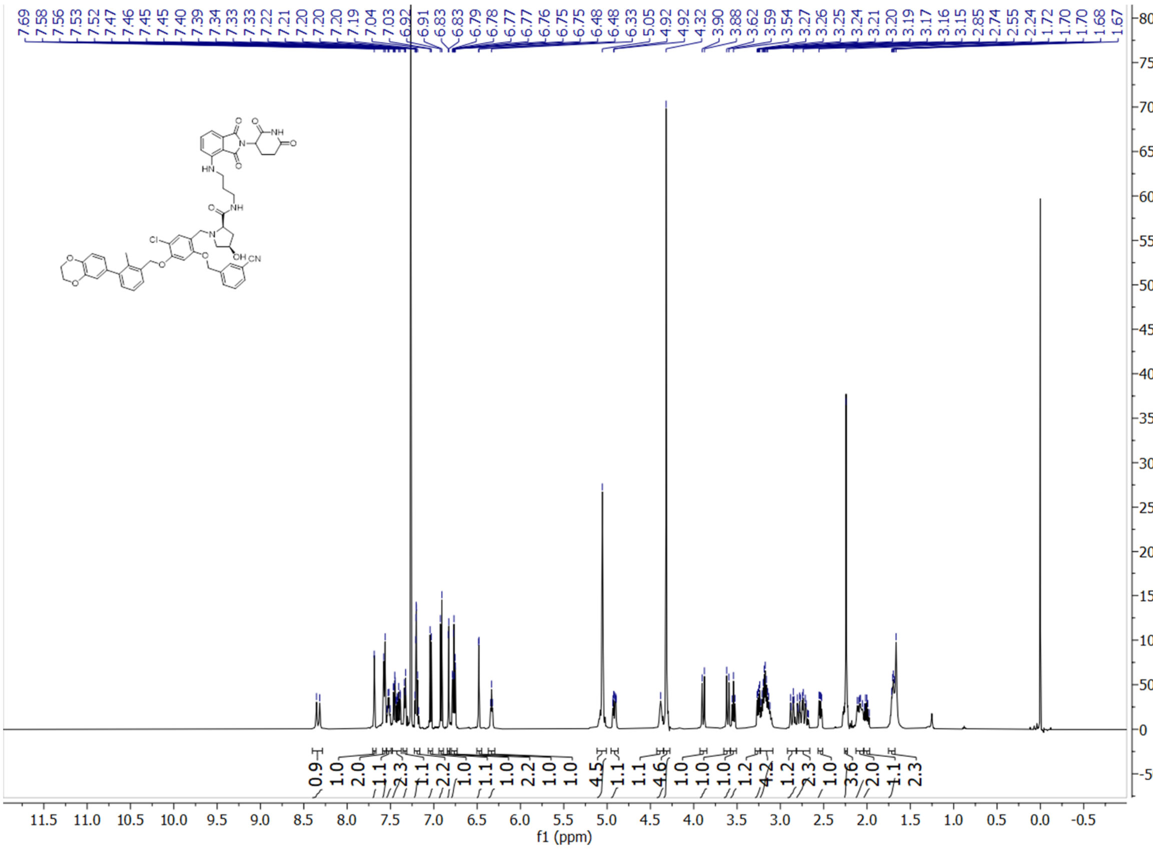

.

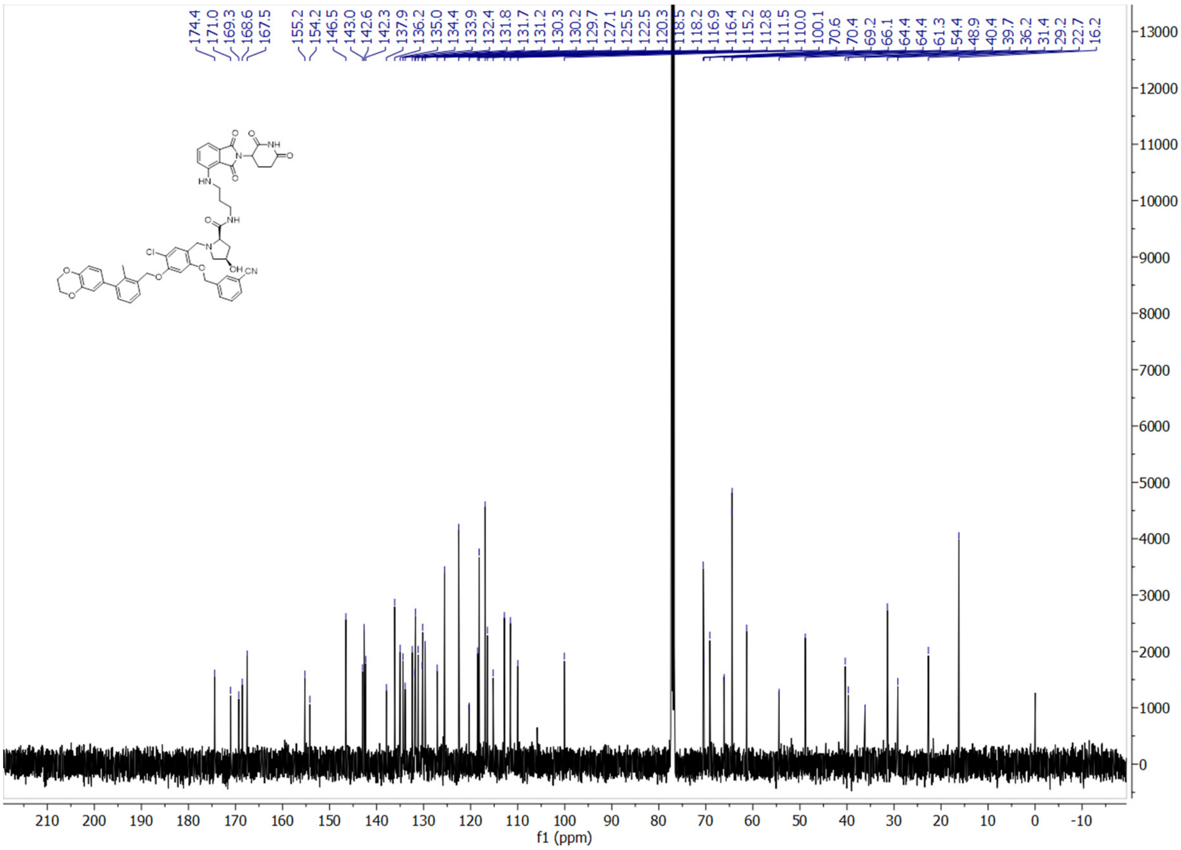

Figure S2. (2R,4R)-1-(5-chloro-2-((3-cyanobenzyl)oxy)-4-((3-(2,3-dihydrobenzo[b][1,4]dioxin-6-yl)-2-methylbenzyl)oxy)benzyl)-N-(3-((2-(2,6-dioxopiperidin-3-yl)-1,3-dioxoisoindolin-4-yl)amino)propyl)-4-hydroxypyrrolidine-2-carboxamide (**2**).

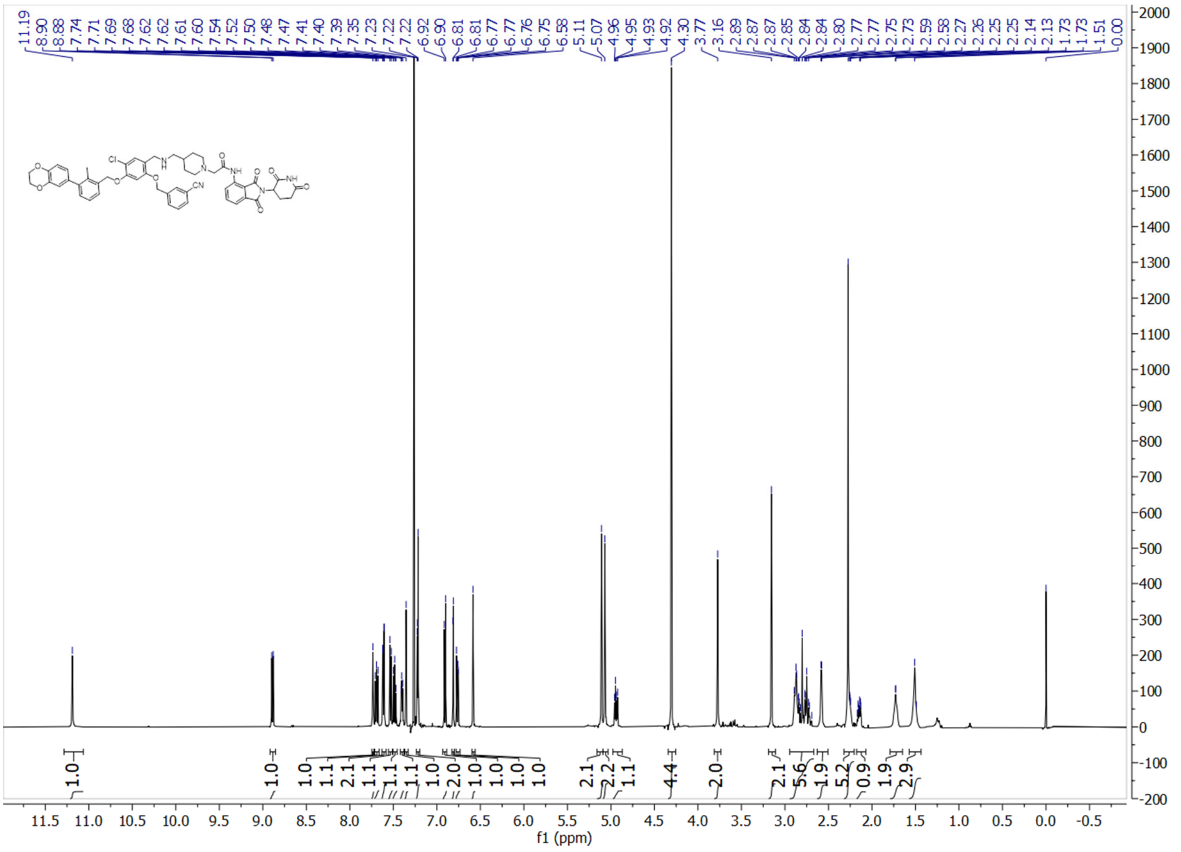

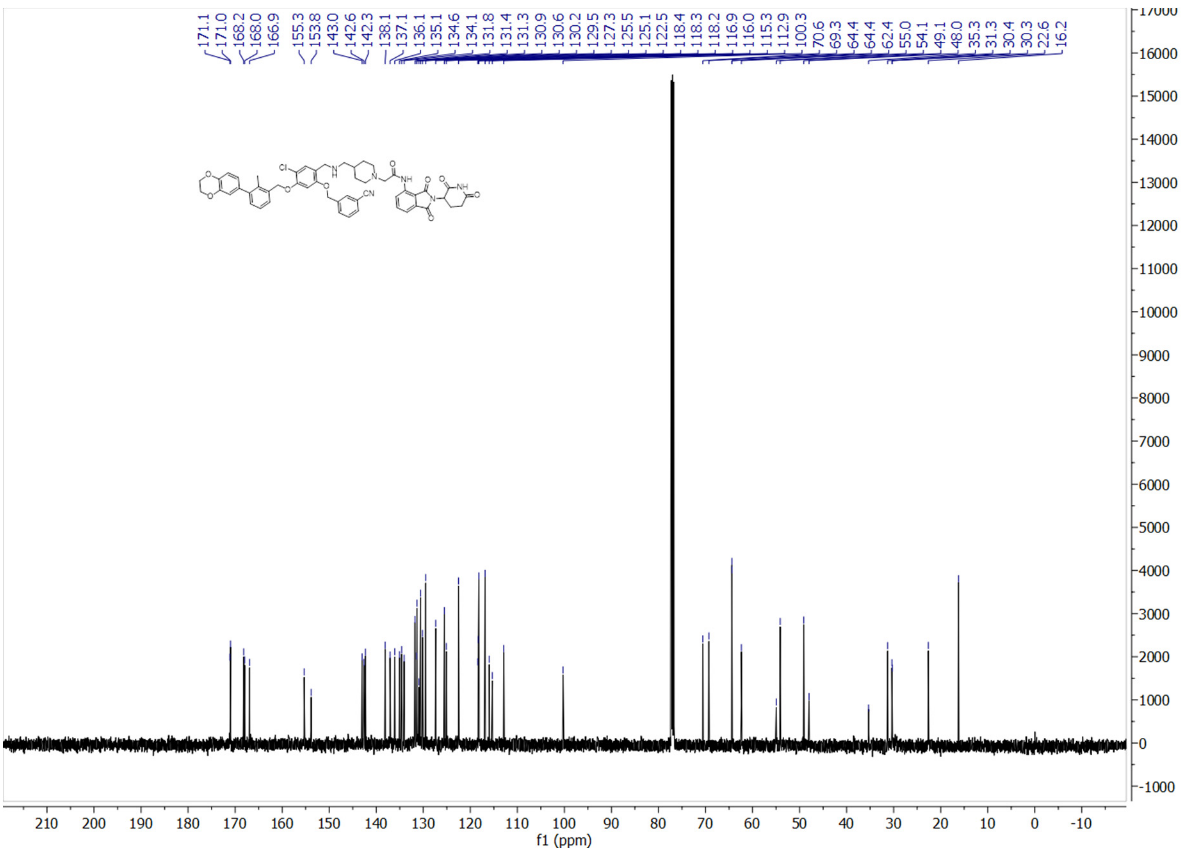

Figure S3. 2-(4-(((5-chloro-2-((3-cyanobenzyl)oxy)-4-((3-(2,3-dihydrobenzo[b][1,4]dioxin-6-yl)-2-methylbenzyl)oxy)benzyl)amino)methyl)piperidin-1-yl)-N-(2-(2,6-dioxopiperidin-3-yl)-1,3-dioxoisoindolin-4-yl)acetamide (**3**).

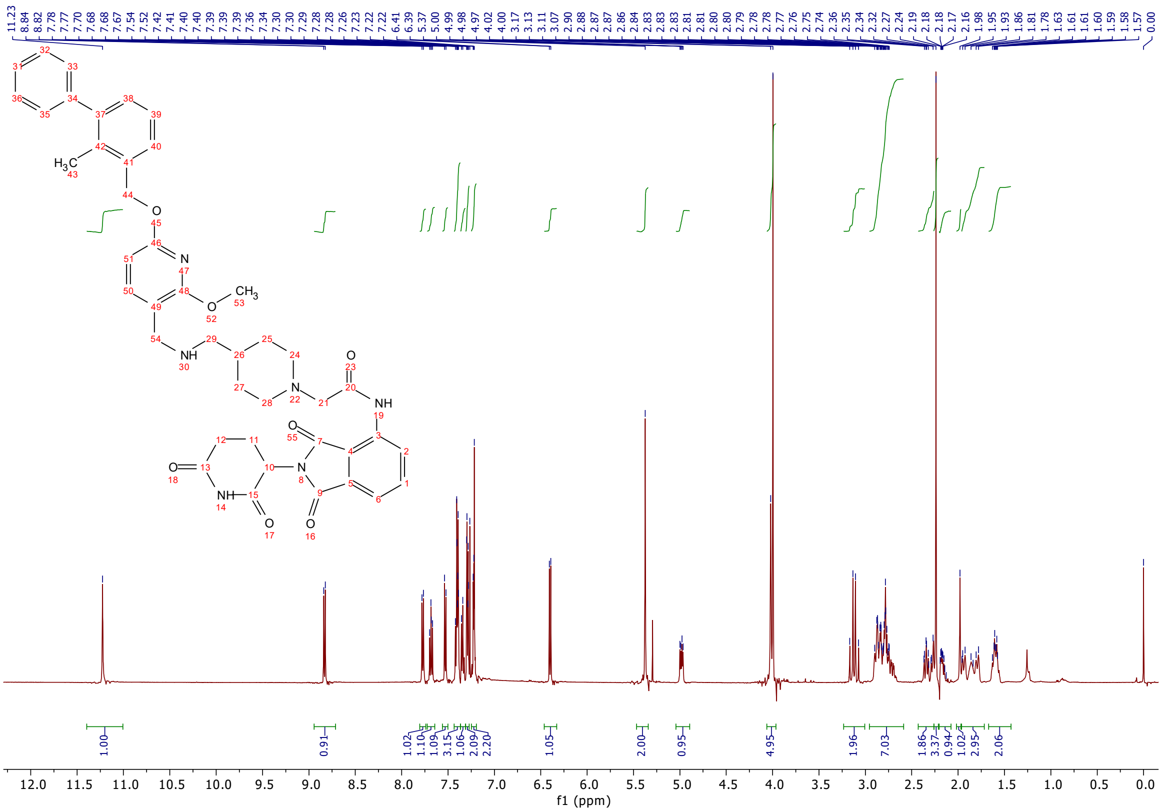

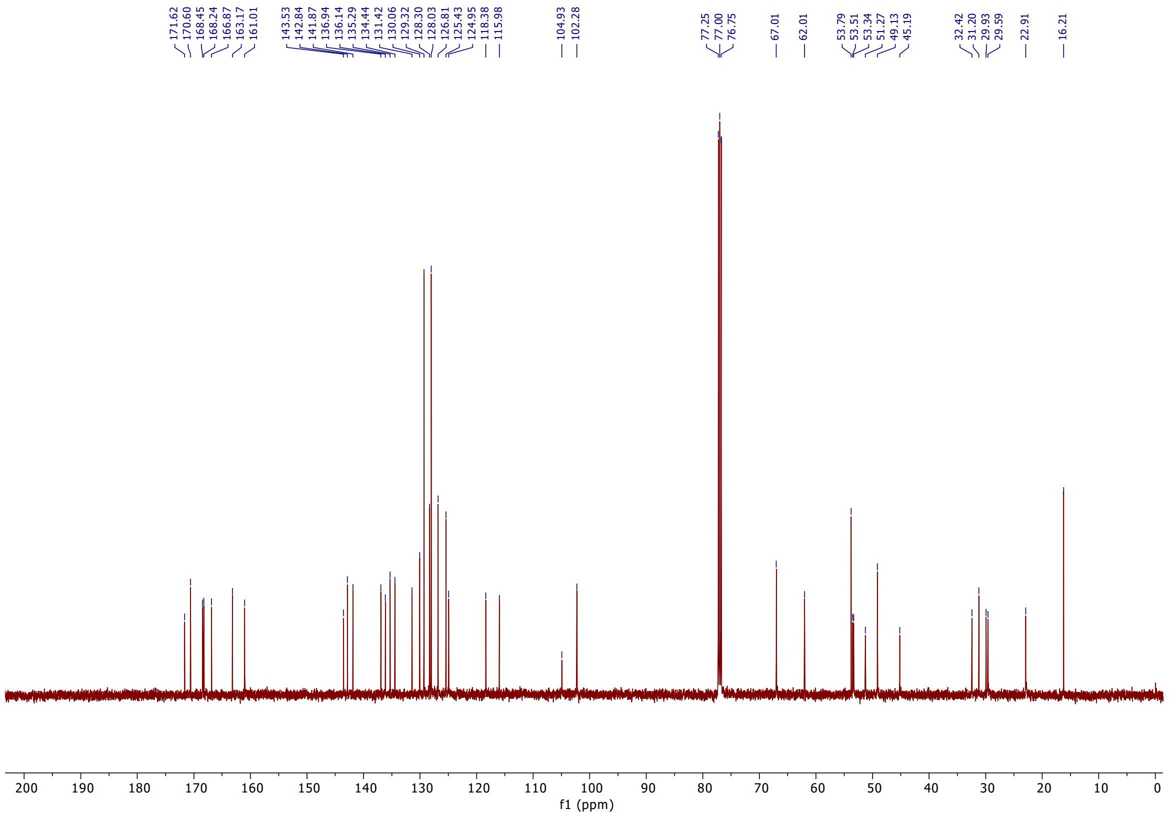

Figure S4. N-(2-(2,6-dioxopiperidin-3-yl)-1,3-dioxoisoindolin-4-yl)-2-(4-((((2-methoxy-6-((2-methyl-[1,1'-biphenyl]-3-yl)methoxy)pyridin-3-yl)methyl)amino)methyl)piperidin-1-yl)acetamide (**4**).

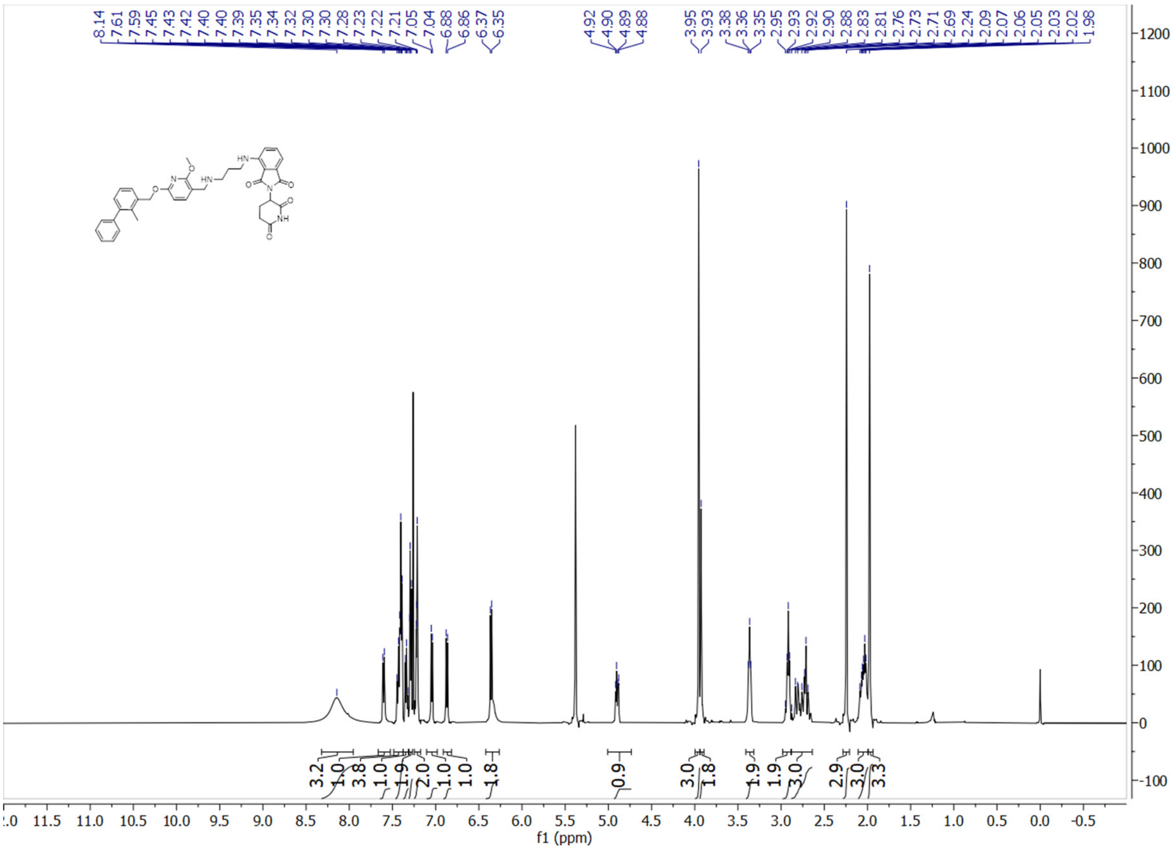

Figure S5. 2-(2,6-dioxopiperidin-3-yl)-4-((3-(((2-methoxy-6-((2-methyl-[1,1'-biphenyl]-3-yl)methoxy)pyridin-3-yl)methyl)amino)propyl)amino)isoindoline-1,3-dione (**5**).

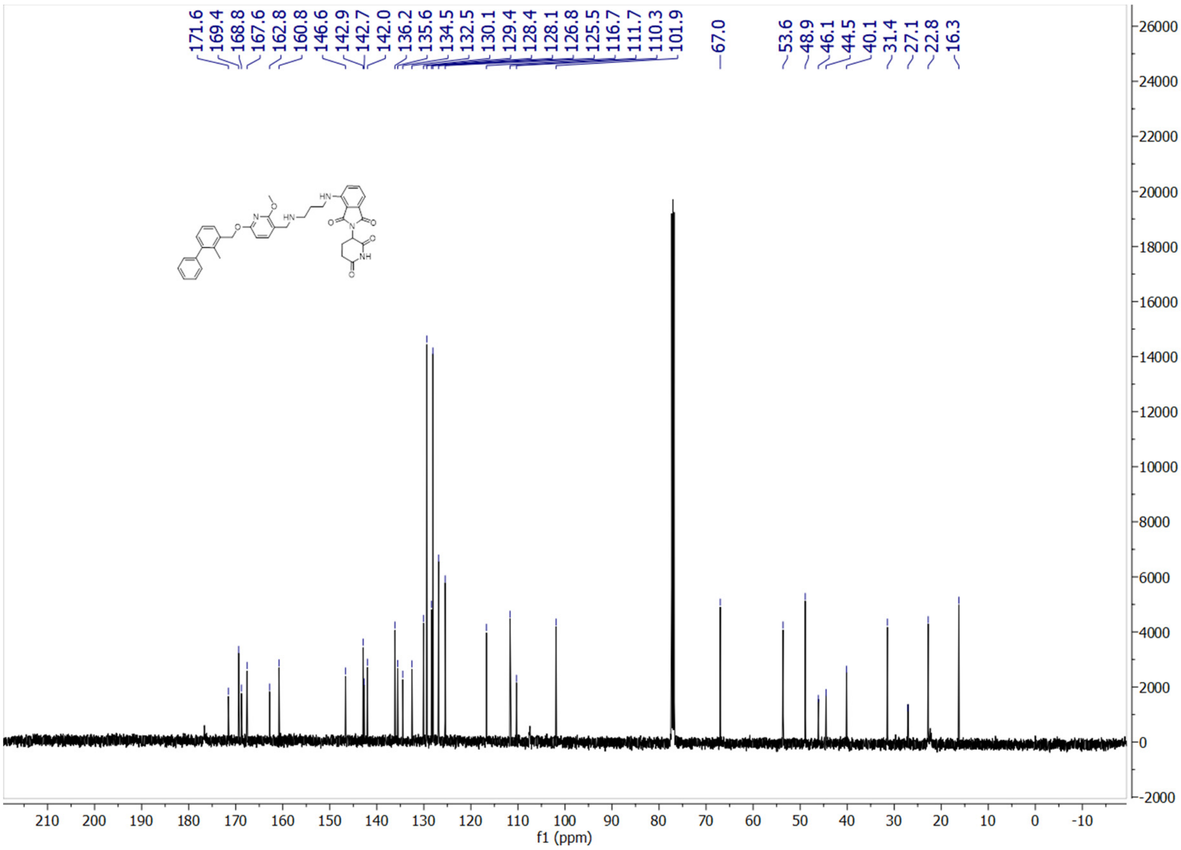

Figure S6. 2-(2,6-dioxopiperidin-3-yl)-4-((3-(((2-methoxy-6-((2-methyl-[1,1'-biphenyl]-3-yl)methoxy)pyridin-3-yl)methyl)amino)propyl)amino)isoindoline-1,3-dione (**5**).

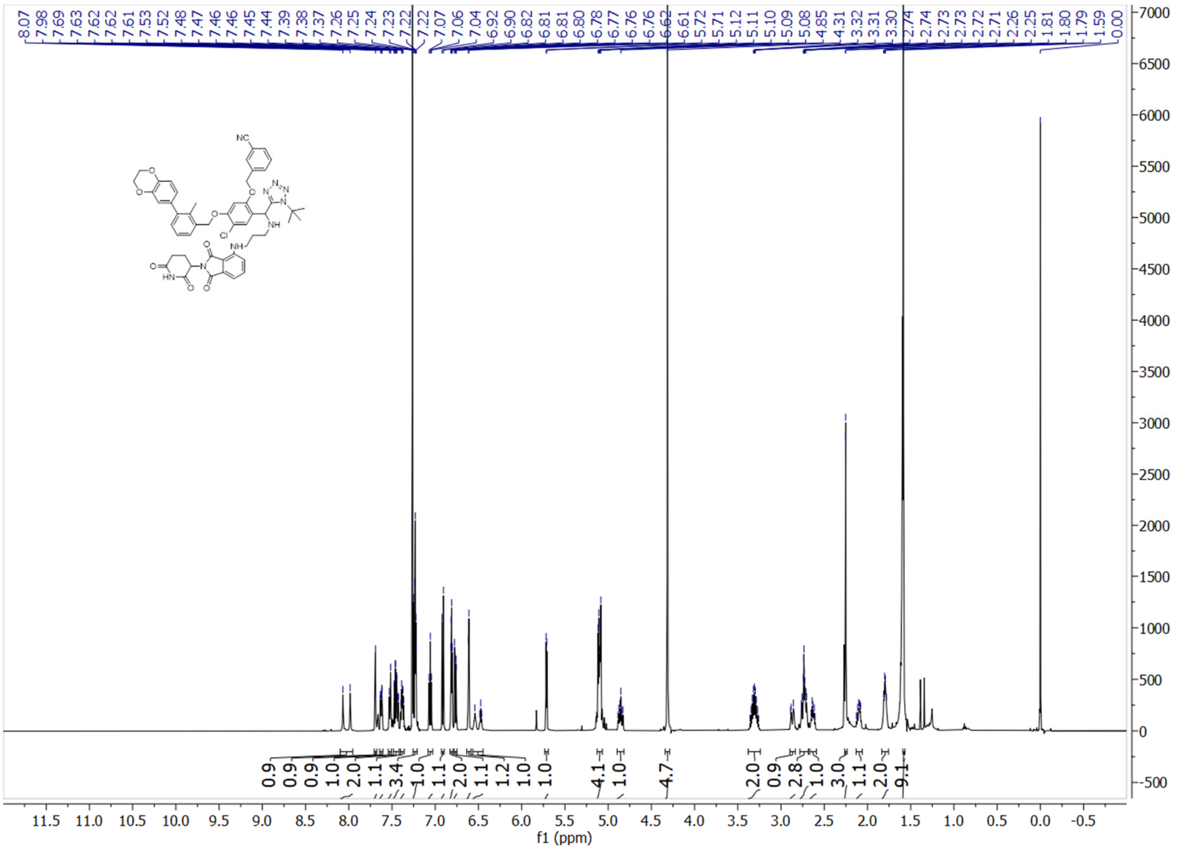

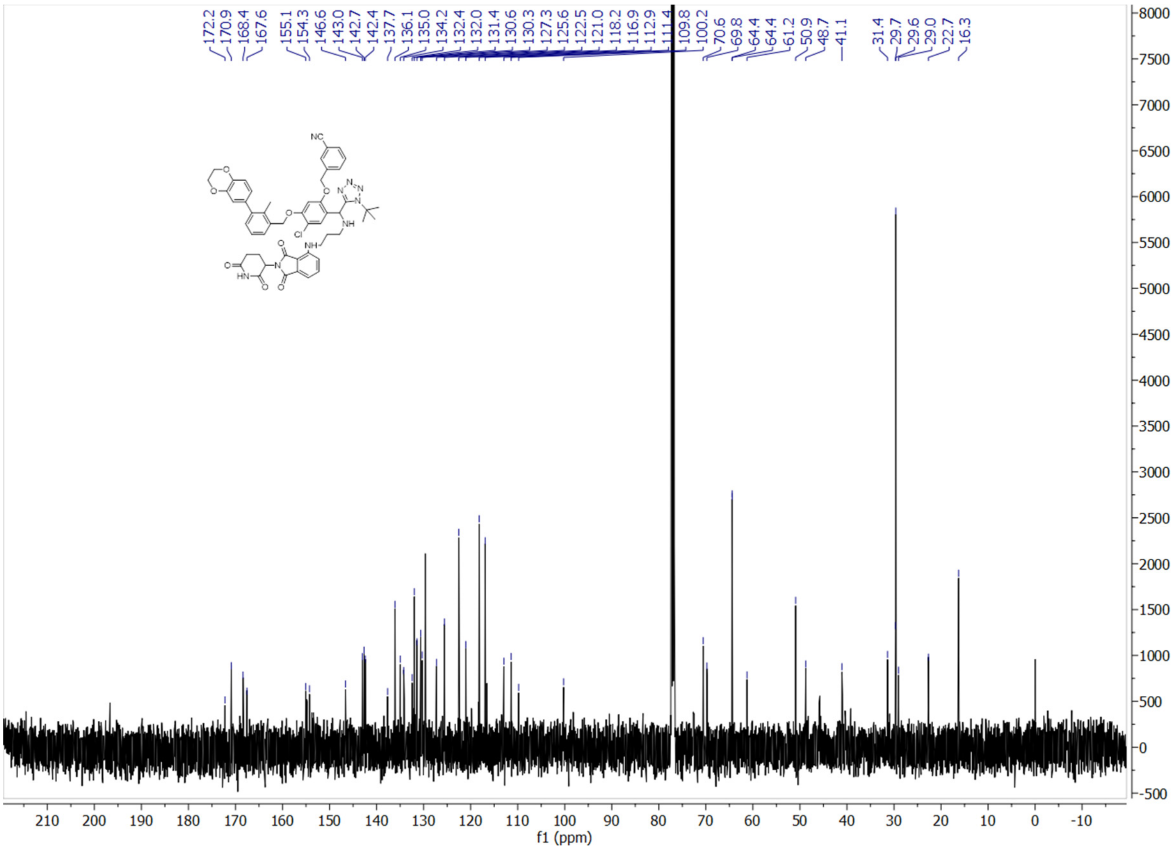

Figure S7. 3-((2-((1-(tert-butyl)-1H-tetrazol-5-yl)((3-((2-(2,6-dioxopiperidin-3-yl)-1,3-dioxoisoindolin-4-yl)amino)propyl)amino)methyl)-4-chloro-5-((3-(2,3-dihydrobenzo[b][1,4]dioxin-6-yl)-2-methylbenzyl)oxy)phenoxy)methyl)benzonitrile (**6**).

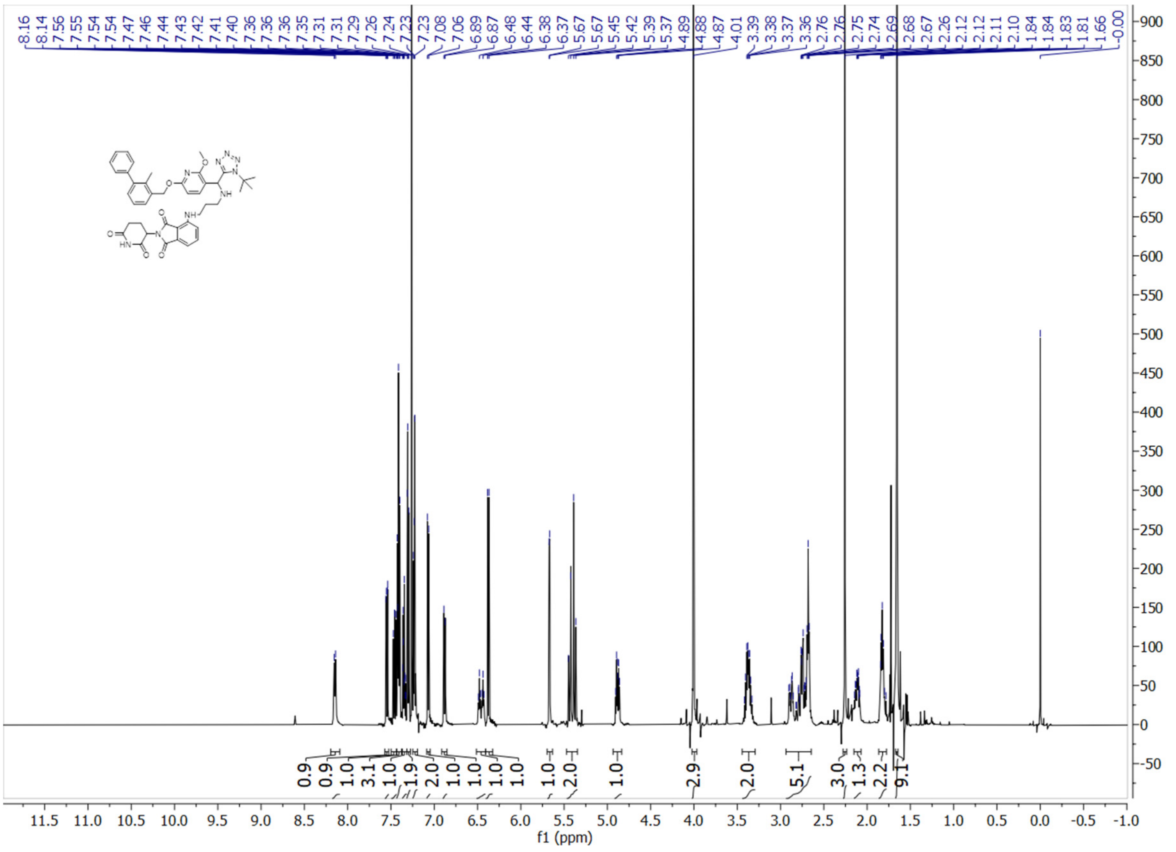

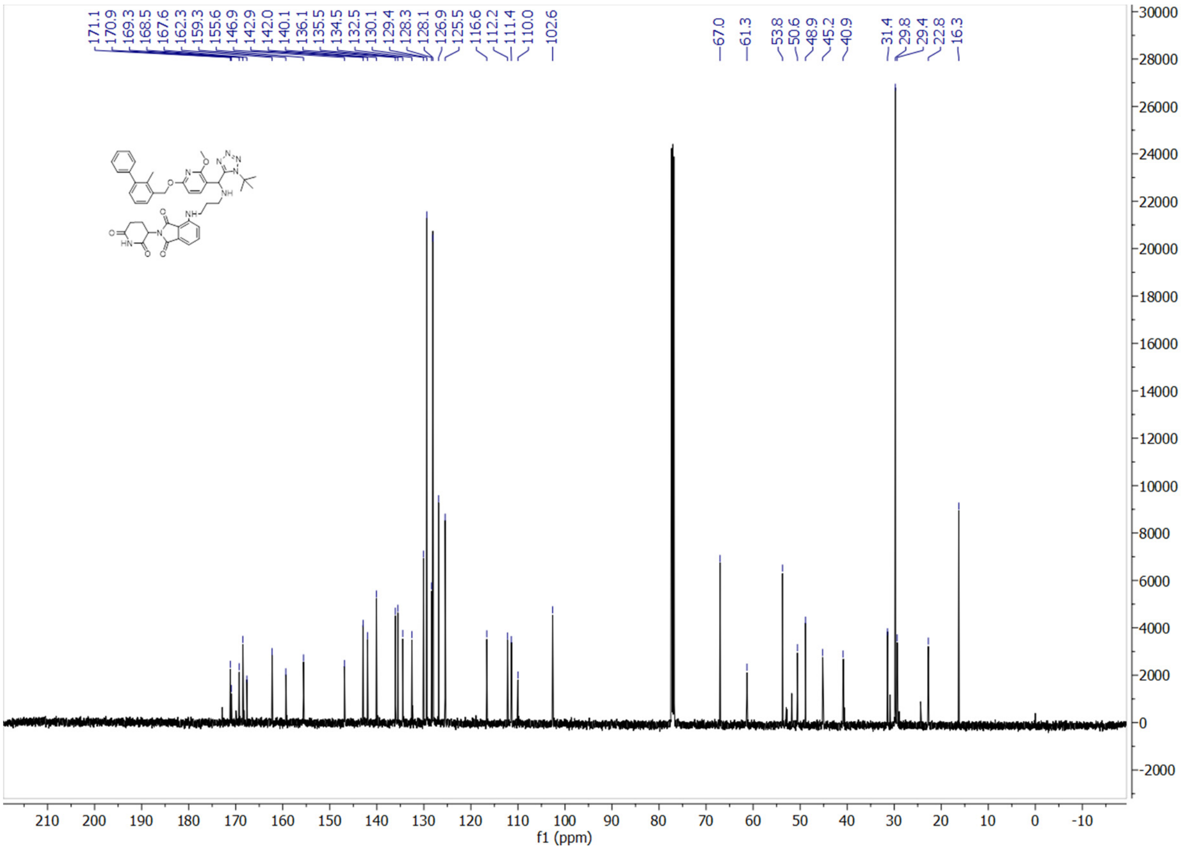

Figure S8. 4-((3-(((1-(tert-butyl)-1H-tetrazol-5-yl)(2-methoxy-6-((2-methyl-[1,1'-biphenyl]-3-yl)methoxy)pyridin-3-yl)methyl)amino)propyl)amino)-2-(2,6-dioxopiperidin-3-yl)isoindoline-1,3-dione (**7**).

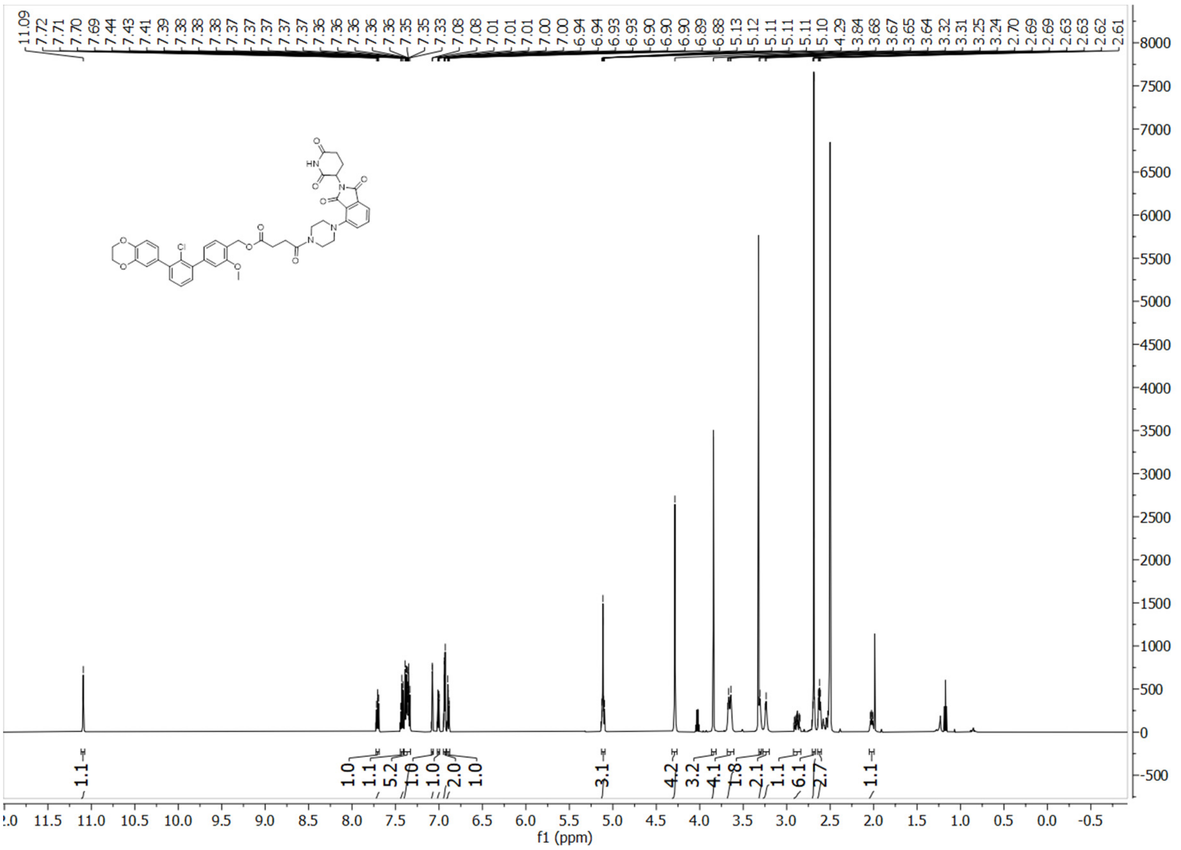

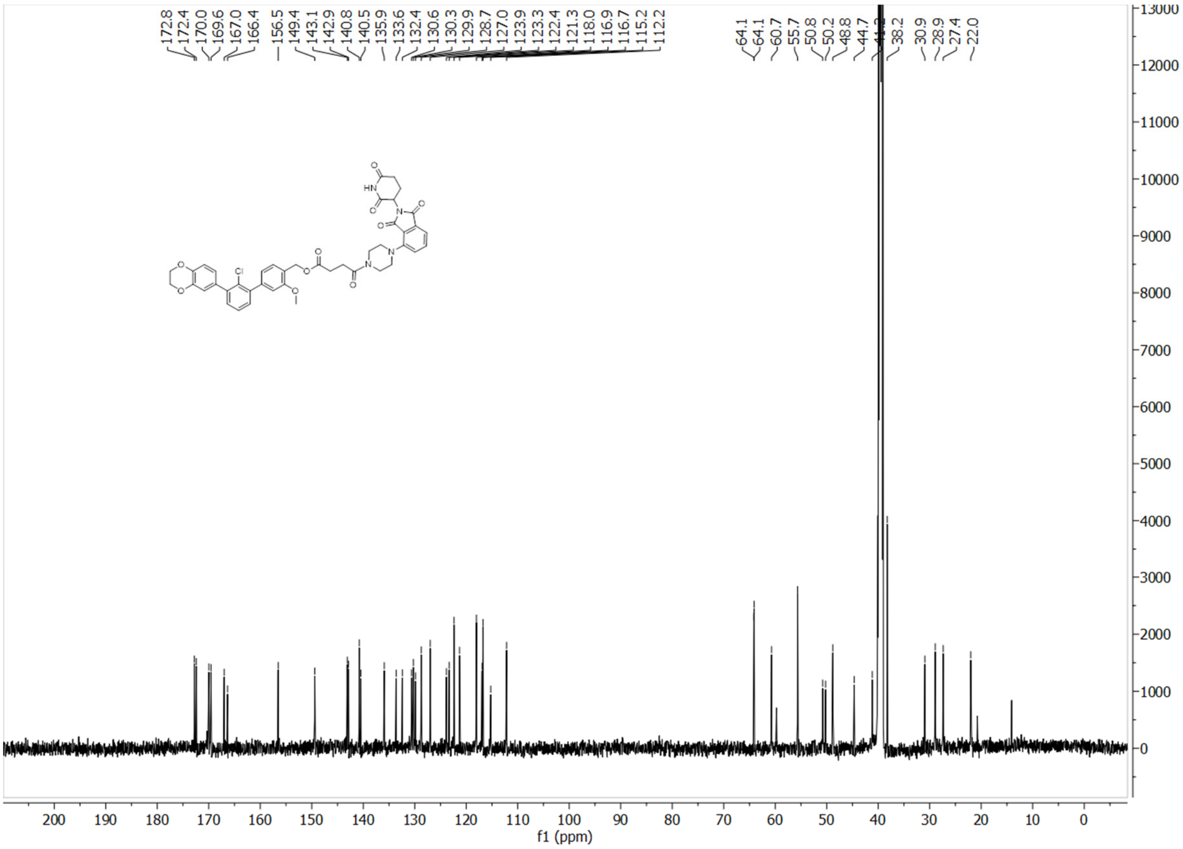

Figure S9. (3'-(benzo-1,4-dioxan-6-yl)-2'-chloro-3-methoxy-[1,1'-biphenyl]-4-yl)methyl 4-(4-(2-(2,6-dioxopiperidin-3-yl)-1,3-dioxoisoindolin-4-yl)piperazin-1-yl)-4-oxobutanoate (**10**).

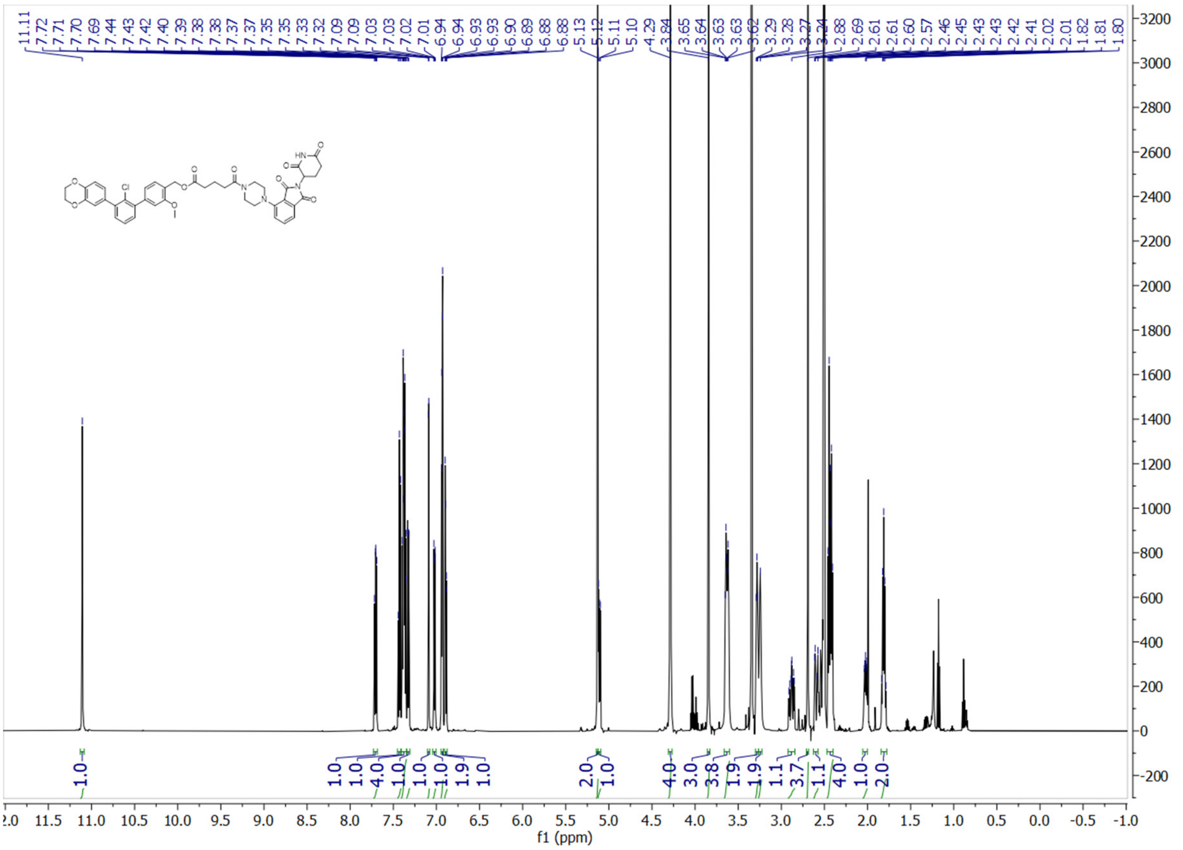

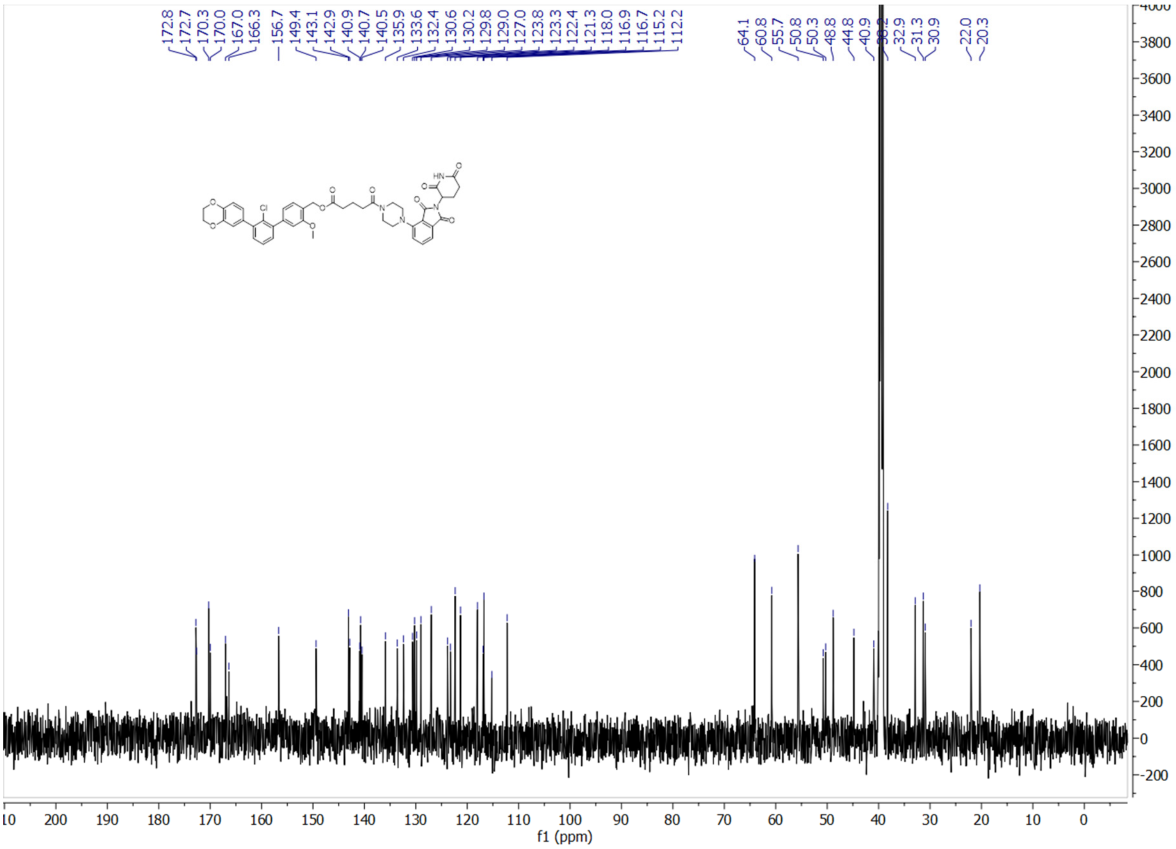

Figure S10. (3'-(benzo-1,4-dioxan-6-yl)-2'-chloro-3-methoxy-[1,1'-biphenyl]-4-yl)methyl 4-(4-(2-(2,6-dioxopiperidin-3-yl)-1,3-dioxoisoindolin-4-yl)piperazin-1-yl)-4-oxopentanoate (**11**).

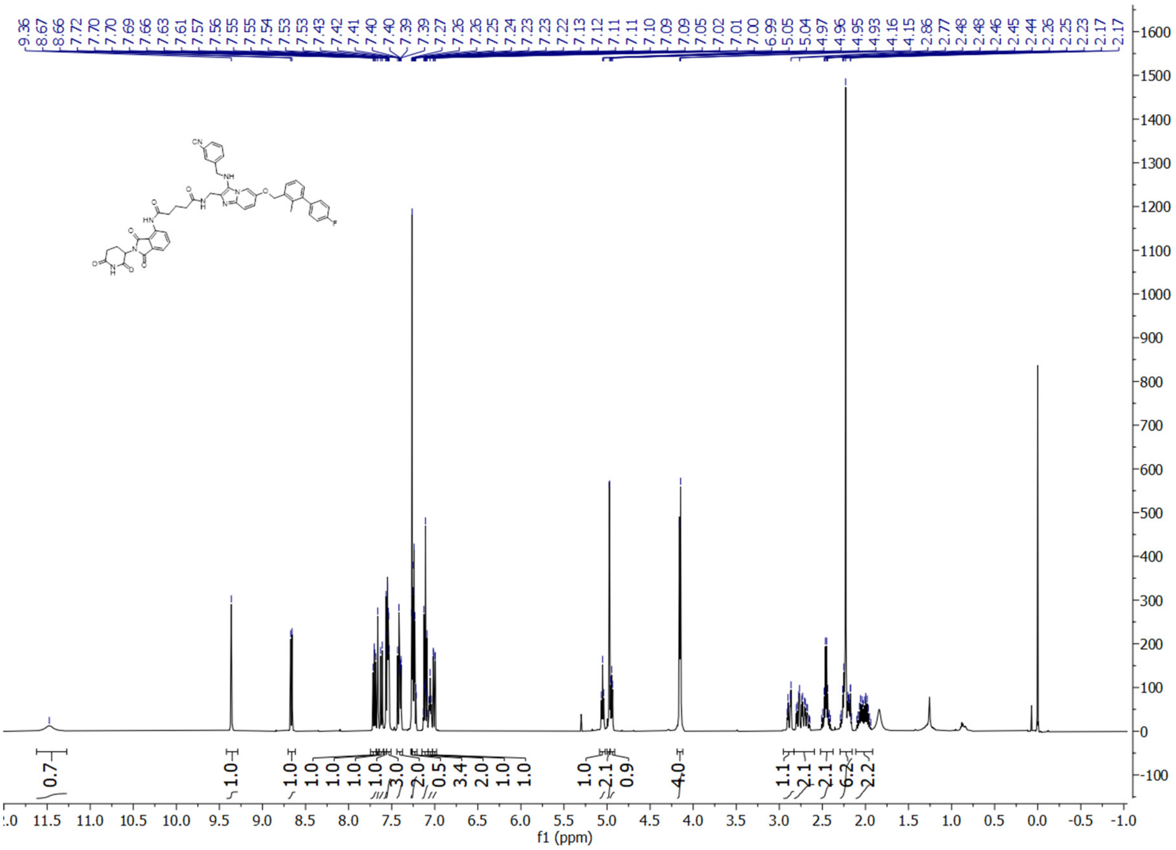

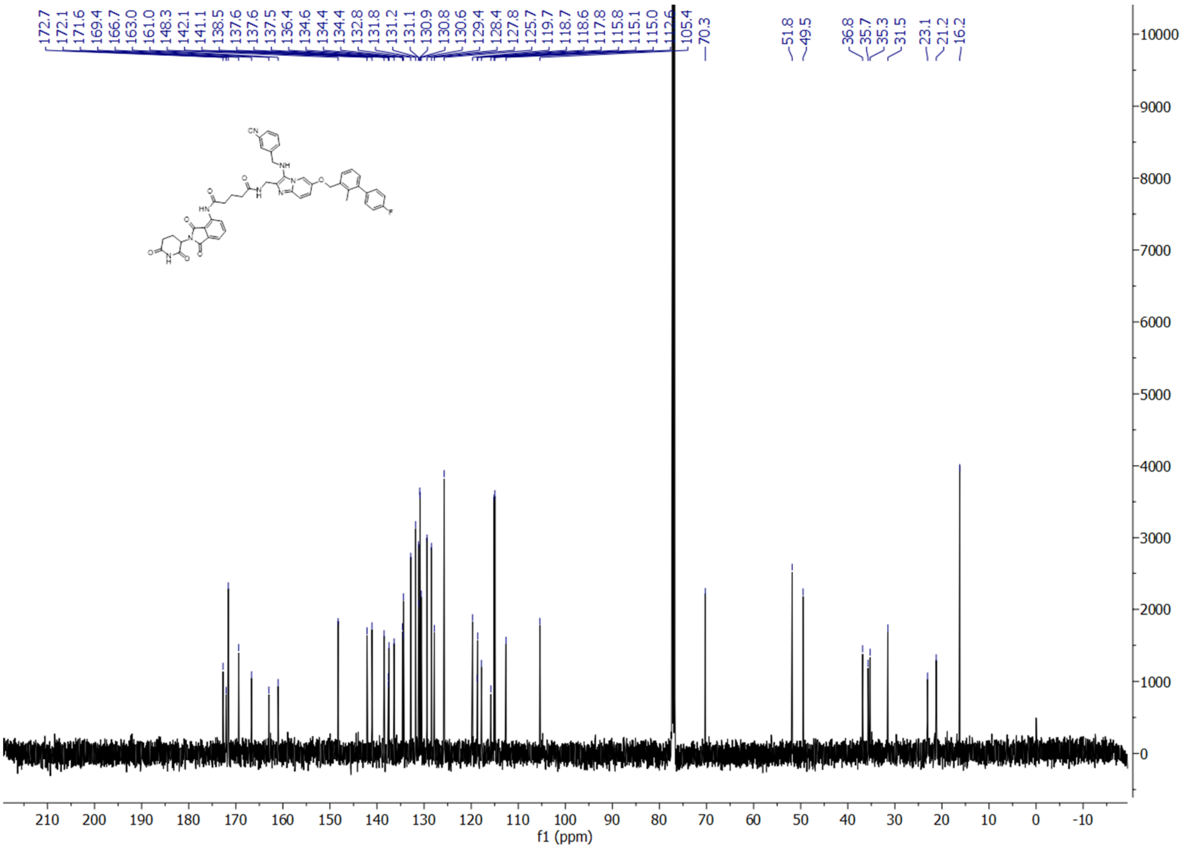

Figure S11. N1-((3-((3-cyanobenzyl)amino)-6-((4'-fluoro-2-methyl-[1,1'-biphenyl]-3-yl)methoxy)imidazo[1,2-a]pyridin-2-yl)methyl)-N5-(2-(2,6-dioxopiperidin-3-yl)-1,3-dioxoisoindolin-4-yl)glutaramide (**12**).

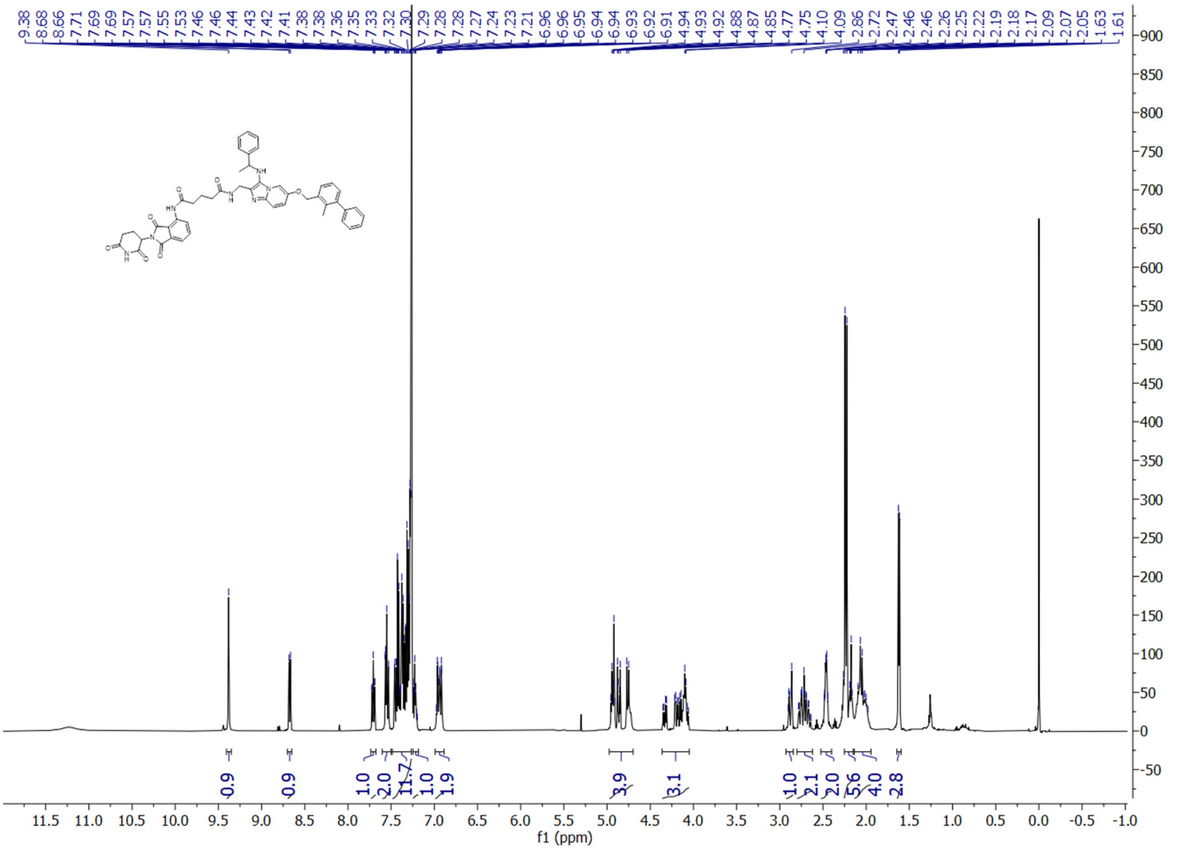

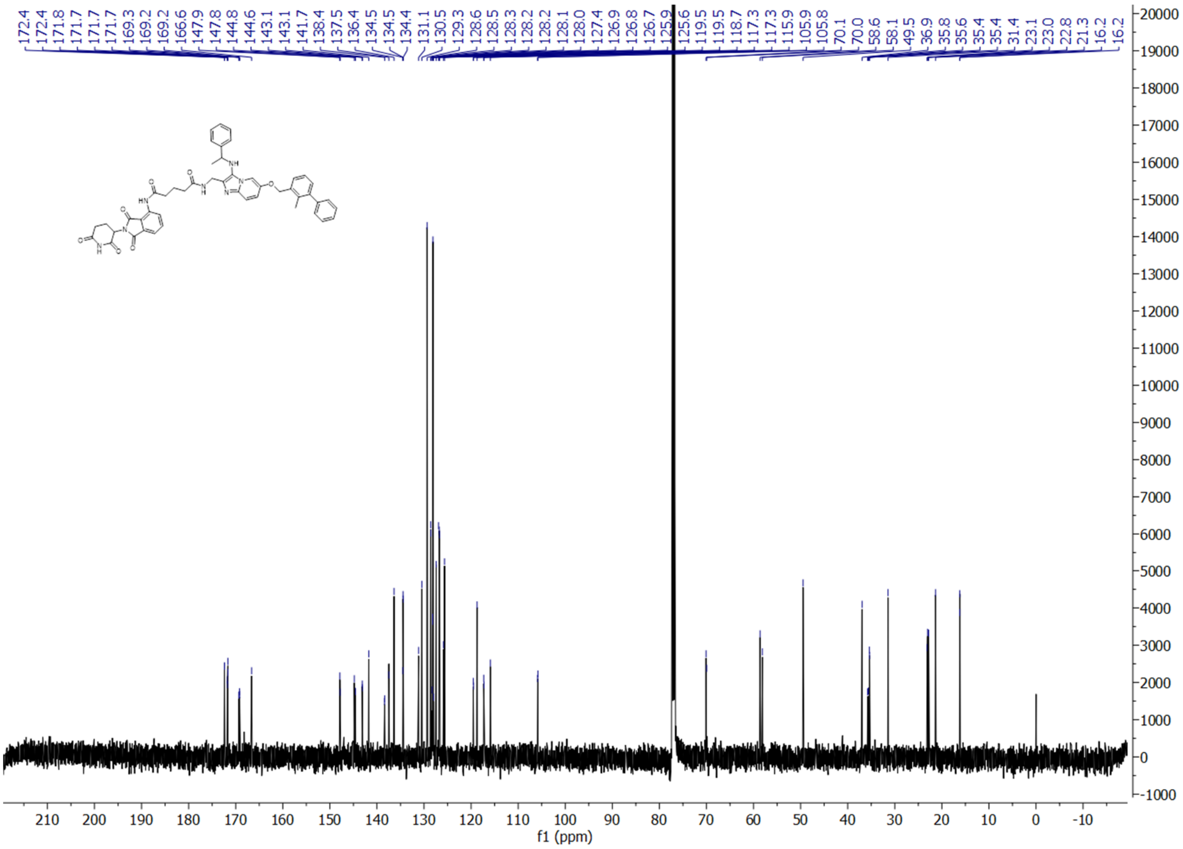

Figure S12. N1-(2-(2,6-dioxopiperidin-3-yl)-1,3-dioxoisoindolin-4-yl)-N5-((6-((2-methyl-[1,1'-biphenyl]-3-yl)methoxy)-3-((1-phenylethyl)amino)imidazo[1,2-a]pyridin-2-yl)methyl)glutaramide (**13**).
